# Metabolic reprogramming is associated with flavopiridol resistance in prostate cancer DU145 cells

**DOI:** 10.1038/s41598-017-05086-6

**Published:** 2017-07-11

**Authors:** Xiaoran Li, Jie Lu, Quancheng Kan, Xiaoli Li, Qiong Fan, Yaqing Li, Ruixia Huang, Ana Slipicevic, Hiep Phuc Dong, Lars Eide, Junbai Wang, Hongquan Zhang, Viktor Berge, Mariusz Adam Goscinski, Gunnar Kvalheim, Jahn M. Nesland, Zhenhe Suo

**Affiliations:** 1 Department of Pathology, The Norwegian Radium Hospital, Oslo University Hospital, Oslo, 0379 Norway; 2 Department of Pathology, Institute of Clinical Medicine, Faculty of Medicine, University of Oslo, Oslo, 0318 Norway; 3 Department of Epidemiology and Biostatistics, College of Public Health, Zhengzhou University, Zhengzhou, Henan 450001 China; 4 Department of Clinical Pharmacology, The First Affiliated Hospital of Zhengzhou University, Zhengzhou, Henan 450001 China; 5 Department of Oncology, The First Affiliated Hospital of Zhengzhou University, Zhengzhou University, Zhengzhou, Henan 450052 China; 6 Department of Nutrition, Institute of Basic Medical Sciences, University of Oslo, Oslo, 0316 Norway; 7 Department of Radiation Biology, Institute for Cancer Research, Oslo University Hospital, Oslo, 0379 Norway; 8 Department of Medical Biochemistry, University of Oslo and Oslo University Hospital, Oslo, 0372 Norway; 9 Laboratory of Molecular Cell Biology and Tumor Biology, Department of Anatomy, Histology and Embryology, Peking University Health Science Center, Beijing, 100191 China; 10 Department of Urology, The Norwegian Radium Hospital, Oslo University Hospital, Oslo, 0379 Norway; 11 Departments of Surgery, The Norwegian Radium Hospital, Oslo University Hospital, Institute for Clinical Medicine, Faculty of Medicine, University of Oslo, Oslo, 0379 Norway; 12 Department of Cell Therapy, Cancer Institute, The Norwegian Radium Hospital, Oslo University Hospital, Oslo, 0379 Norway

## Abstract

Flavopiridol (FP) is a pan-cyclin dependent kinase inhibitor, which shows strong efficacy in inducing cancer cell apoptosis. Although FP is potent against most cancer cells *in vitro*, unfortunately it proved less efficacious in clinical trials in various aggressive cancers. To date, the molecular mechanisms of the FP resistance are mostly unknown. Here, we report that a small fraction human prostate cancer DU145 cells can survive long-term FP treatment and emerge as FP-resistant cells (DU145^FP^). These DU145^FP^ cells show accumulated mitochondrial lesions with stronger glycolytic features, and they proliferate in slow-cycling and behave highly migratory with strong anti-apoptotic potential. In addition, the cells are less sensitive to cisplatin and docetaxel-induced apoptotic pressure, and over-express multiple stem cell associated biomarkers. Our studies collectively uncover for the first time that FP-resistant prostate cancer cells show metabolic remodeling, and the metabolic plasticity might be required for the FP resistance-associated cancer cell stemness up-regulation.

## Introduction

Human neoplasms consist of heterogeneous cell subtypes, and some of the minor cell populations have specific features, and are often referred to as cancer stem cells (CSCs)^1, 2^. It is known that CSCs have capacity of self-renewal, differentiation potential, and are highly tumorigenic and resistant to conventional therapies^3, 4^. Many current studies have suggested that CSCs are the driving force of tumorigenesis, and precise elimination of CSCs present a favorable way of targeting cancers^5, 6^. However, studying CSCs is difficult, since they can be so diverse from one tumor to another^7^ while often being rare in a given tumor and difficult to be purified *in vitro* ^8, 9^.

It is known that CSCs often express specific molecular markers such as integrin α2β1, CD44, CD133, CXCR4, ABCG2, ALDH2 and ALDH1A1^5, 10^. These molecules have been correlated with cancer metastasis and multidrug resistance, which represent the main reasons for cancer treatment failure^11^. Previously, it has been demonstrated that various anti-cancer therapies can promote emergence of CSC-like cell subpopulations^5, 12, 13^, suggesting a possibility that therapeutic agents can be applied to select and enrich CSC-like cells *in vitro*. Among many new drugs developed for cancer targeted therapies in recent decades, cyclin dependent kinase (CDK) inhibitors have shown unique advantages in targeting various cancers, e.g. by intercepting their dysregulated cell cycle, and thus have become potential candidates for clinical cancer treatments^14–16^. As one of the best studied compounds, flavopiridol (FP) is the first CDK inhibitor to undergo a phase I/II evaluation in clinical trials^17, 18^. Besides its capability to eliminate proliferating cells, FP is also efficacious against dormant cancer cells, even in those which have deficient pRb and/or p53 gene products^19–22^. FP is a synthetic flavone derived from flavonoid rohitukine and shows a strong capacity to inhibit phosphokinases^23^. Its activity is strongest among CDKs, but FP also inhibits a series of other kinases such as EGFR, Src, Lck, PKA and PKC etc.^24^. As another important anti-cancer activity, FP has been reported resulting in fast mitochondrial lesions and ER stress, which finally lead to apoptosis and autophagy^25–27^. Although FP performs diverse anti-tumor activities, unfortunately, like other chemotherapeutic agents, the FP clinical application fails to show significant efficacy because of treatment resistance, and the mechanisms behind this are largely unexplored.

In the current study, we treated the DU145 prostate cancer cells with 400 nM FP and discovered that after an initial induction of apoptosis in majority of the cells, less than 1% of the DU145 cells survived and underwent an adaptation process of about 46 days. These cells slowly resumed proliferation and emerged as a FP-tolerant cell line (DU145^FP^ line). Our data demonstrates that the DU145^FP^ cells are slow cycling and frequently show mitochondrial depolarization. The DU145^FP^ cells also produce abundant anti-apoptotic proteins and display reduced sensitivity to other therapeutic drugs-induced apoptotic pressure such as docetaxel. Besides that, the DU145^FP^ cells show altered cell metabolism preferences, which are reflected by the enhanced glucose uptake, reduced oxygen consumption and stronger extracellular acidification ratio, collectively known as the Warburg effect. Furthermore, the DU145^FP^ cells display advanced neoplastic aggressiveness, since the cells show greater cell motility and highly express a series of cancer stemness related proteins. The present study provides sufficient evidence that FP induced mitochondrial lesions can initiate cell metabolism remodeling and up-regulate cancer cell stemness, highlighting potential contributions of the mechanisms in FP treatment failure.

## Results

### A small sub-population of DU145 cells have survived the long-term FP treatment

**Figure 1:**
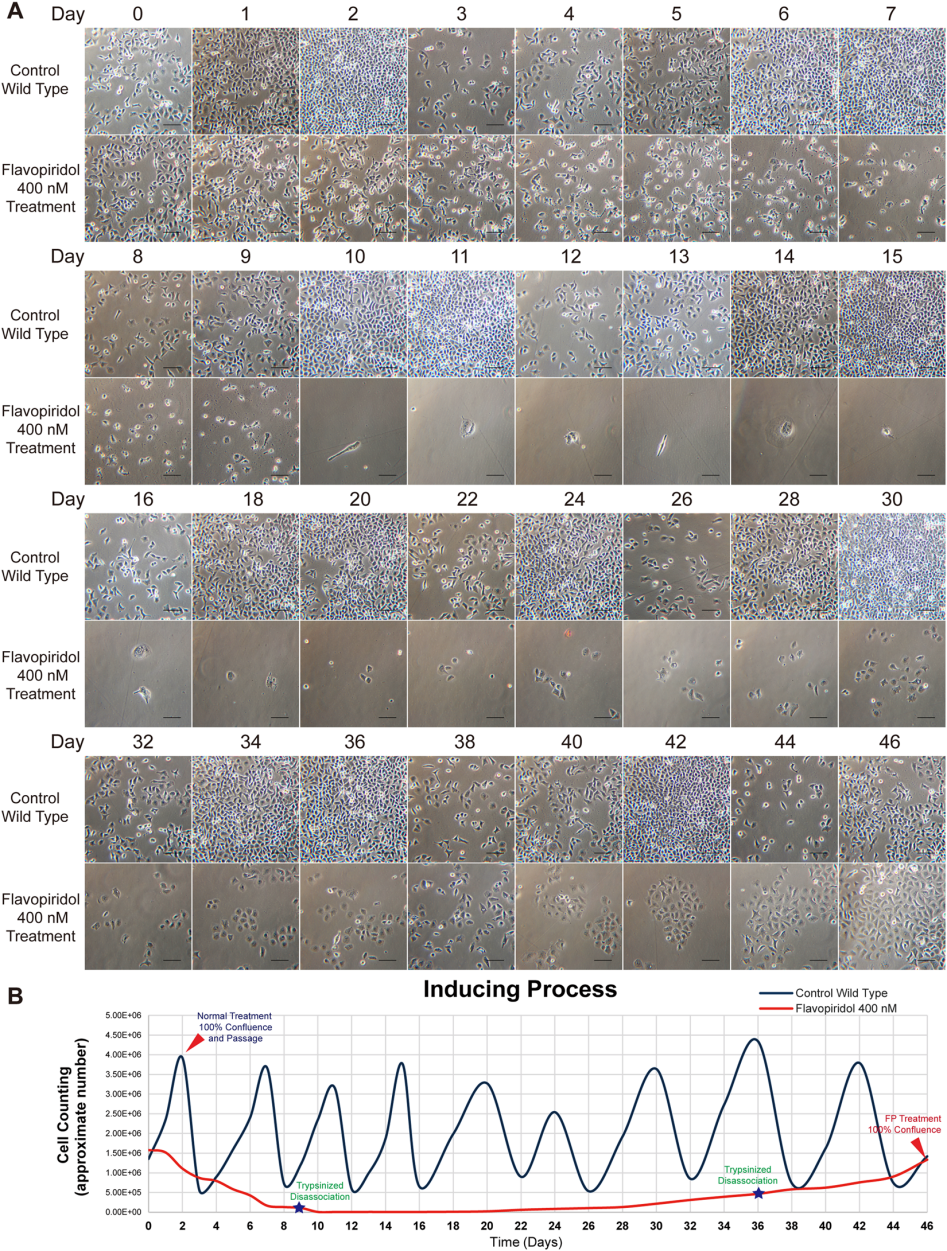
DU145 cells during the FP treatment adaptation process. (**A**) FP-resistant DU145 cells are developed through a 46 days’ adaptation process. The wild type DU145 cells are displayed as controls in the upper rows, and the DU145 cells exposed to 400 nM FP are shown in the lower rows. The scale bar equals to 100 µm. (**B**) Line charts display the typical adaptation process. The dark blue- and red lines denote the approximate cell count fluctuations in a period of 46 days. The dark blue line represents the wild type DU145 cells, while the red line denotes the status when DU145 cells were treated with FP. The blue peaks denote each passage of DU145^WT^ cells, the red triangle represents cell confluence, and the blue stars denote trypsinized dissociation during the 400 nM FP treatment.

To establish FP tolerant DU145 line, we treated the DU145 cells with 400 nM flavopiridol hydrochloride plus 1 mM sodium pyruvate in RPMI-1640 media as supplement, based on our preliminary FP concentration-dependent experiments (data not shown). We observed that a small population of the DU145 cells can survive the FP treatment, and the adaptation to FP conditions required 45–50 days’ period. As shown in Fig. 1A, immediately after being exposed to 400 nM FP, the DU145 cells show sharply suppressed cell proliferation and decreased viability. In short, the overall adaptation process consisted of the following three stages: The first induction stage embraced the first 10 days. During this period, all cells incapable of surviving the 400 nM FP pressure were eliminated. Since it is known that massive apoptotic death produces fragments which negatively influence the survival of the remaining cells, the culture medium was changed with newly prepared medium daily. The surviving cells were carefully dissociated and transferred into new culture flasks on day 7–9 for further follow-up. The second stage usually began on day 10 and ended approximately on day 20. During this period, the surviving cells exhibited dormant division activity and were frequently found with epithelial-mesenchymal transformation (EMT) features, with greater mobility and as syncytia compared to the wild type DU145 cells (see Videos SV1 and SV2). The third stage started approximately on day 20. From this point the cells started to divide and finally reached confluence approximately on day 44–46. The cells that survived the 400 nM FP treatment gradually recovered their proliferation activity but still in slow-cycling. These FP resistant cells were continuously maintained in the medium containing 400 nM FP. These cells were then referred to as DU145^FP^, while parental wild type cells were termed DU145^WT^. Figure 1B illustrates the adaptation process based on the cell counting results from the daily captured images. To assess whether those DU145^FP^ cells have extended capacities against FP treatment in higher-concentrations, we treated them with 400–4000 nM FP for durations of 24–96 hours. The viable cells were detected by using flow-cytometry every 24 hours, and the cell viabilities were used to evaluate their resistant capacity to FP treatments accordingly. A group of representative results is shown in the Figure S1A and B. The data shows that even it was treated with 10-folds higher FP concentration for 96 hours, those DU145^FP^ cells still held ~60% of viable cells, whereas only ~10% of the wild type cells survived the same condition. These results therefore confirm that the DU145^FP^ cells are indeed FP-resistant.

### DU145^FP^ cells exhibit higher frequency of mitochondrial depolarization

Previous studies have shown that the FP treatment may result in fast mitochondrial depolarization^25, 26^, which is also confirmed in the DU145 cells in our study as shown in Figure S2. However, considering the fact that the effects of long-term FP treatment on mitochondrial membrane potential (Δψ_m_) have not been reported in this prostate cancer cell line, we next investigated the Δψ_m_ status in our DU145^FP^ cells.

**Figure 2:**
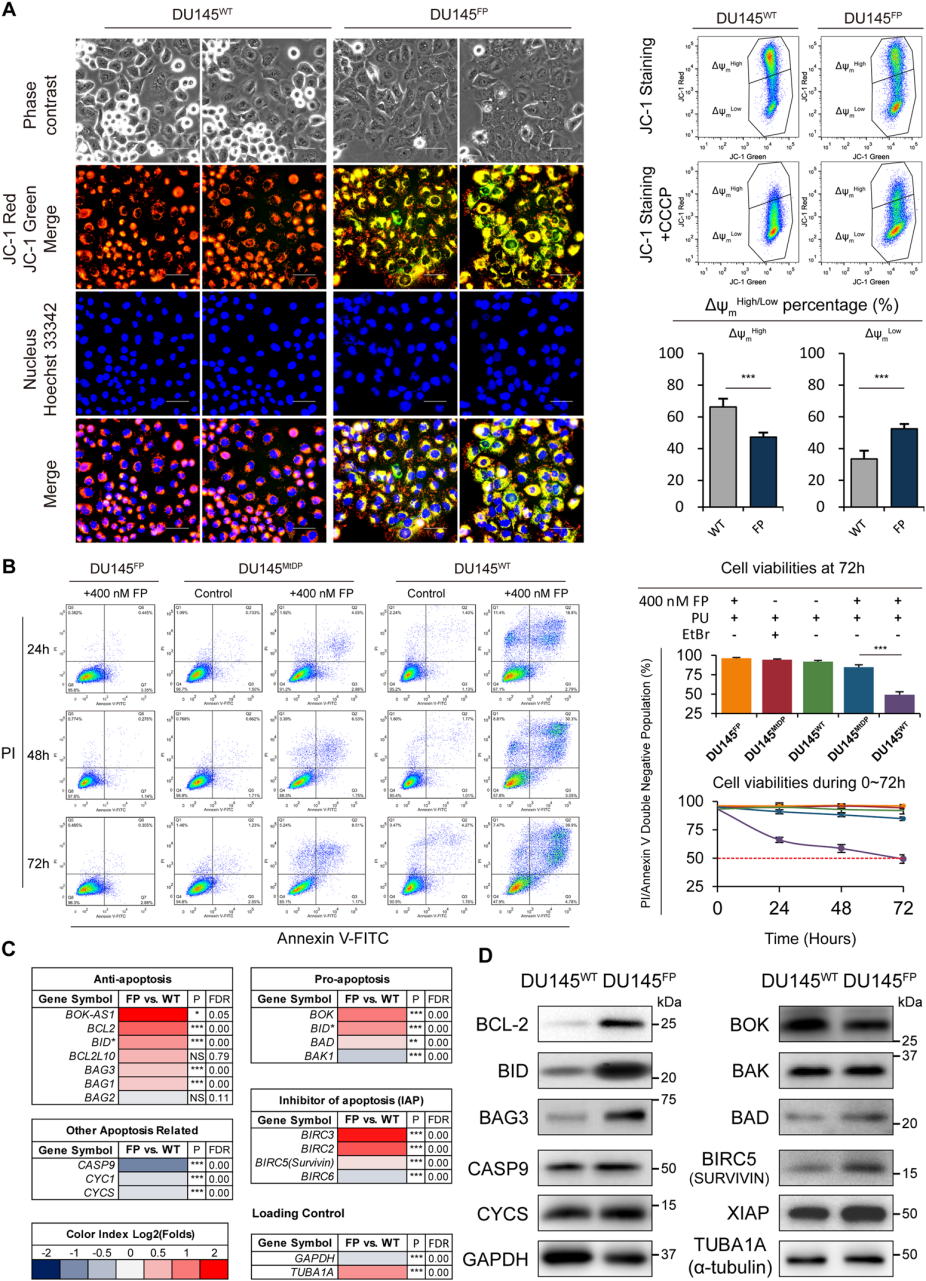
FP-induced DU145 cell death is mitochondria-dependent, and the DU145^FP^ cells express higher levels of anti-apoptotic factors. (**A**) Left: mitochondrial membrane potential is presented by JC-1 probe excitation status. The red signals denote mitochondrial ${{\rm{\Delta }}{\rm{\psi }}}_{{\rm{m}}}^{{\rm{High}}}$ features, whereas green signals denote mitochondrial ${{\rm{\Delta }}{\rm{\psi }}}_{{\rm{m}}}^{{\rm{Low}}}$ features. The abbreviation Ph stands for phase contrast, and the scale bars represent 100 µm. Right: Flow cytometry analyses of Δψ_m_. The cells are stained with JC-1, and CCCP controls were used to confirm that the JC-1 response is sensitive to changes in membrane potential. Based on the high and low Δψ_m_ status, the accordant cell proportions are summarized and shown in the histograms below. The data is presented as means from three independent experiments (mean ± S.D). (**B**) The DU145^FP^, DU145^MtDP^ and DU145^WT^ cells were maintained in corresponding media with or without 400 nM FP for 24–72 hours. The cells’ double negative for both Annexin V-FITC and PI signal are considered as viable. The cell viability is presented in histograms and line charts. The data is presented as means from three independent experiments (mean ± S.D). (**C**) Transcriptome analyses of anti-apoptosis and pro-apoptosis genes. The Log_2_ transformed ratio of FPKM values (e.g., FP/WT) are indicated by color-coded index bars. (**D**) The expression levels of apoptosis-associated proteins were detected by immunoblotting. Statistical significance: *p < 0.05, **p < 0.01, ***p < 0.001.

To measure the Δψ_m_, we stained the DU145^WT^ and DU145^FP^ cells with Δψ_m_ sensitive probe JC-1. The signal of JC-1 aggregates is considered as the marker of mitochondrial polarization (JC-1 red) whereas the cells only containing JC-1 monomers indicate mitochondrial depolarization (JC-1 green)^28^. As shown in the left side of Fig. 2A, the DU145^WT^ cells show relatively well-maintained Δψ_m_ since no obvious JC-1 monomer signal (green) was observed in the fluorescence microscopy images. However, the DU145^FP^ cells exhibit significantly higher frequency of mitochondrial depolarization events (green). To further verify these results, we also analyzed the JC-1 staining by flow-cytometry in all cells, and sufficient carbonyl cyanide 3-chlorophenylhydrazone (CCCP) was added to the control staining, since CCCP effectively collapses Δψ_m_ in diverse cells^29^. Representative results are shown in the upper right of Fig. 2A, and the summarized ${{\rm{\Delta }}{\rm{\psi }}}_{{\rm{m}}}^{{\rm{High}}/{\rm{Low}}}$ ratios are shown in the histograms below. The results confirm the microscopic observations and reveal that the DU145^FP^ cells contain ~20% more cells with depolarized mitochondria compare to the DU145^WT^ cells (${{\rm{\Delta }}{\rm{\psi }}}_{{\rm{m}}}^{{\rm{Low}}}$, ~52% vs. ~33%, DU145^FP^ vs. DU145^WT^). Considering that the integrated electron transport chain (ETC) is crucial for maintaining mitochondrial membrane potential, and damaged ETC often leads to mitochondrial depolarization, we explored the ETC related genes’ expression in the DU145^FP^ and DU145^WT^ cells by transcriptome analysis. The data are summarized in Supplemental Figure S3A. Alteration of the ETC-related genes expression was revealed and approximately half of the ETC-related genes were down-regulated. In line with this finding, mitochondrial reactive oxygen species (ROS) level was also significantly elevated in the DU145^FP^ cells as shown in Figure S3B. Increasing levels of ROS have been confirmed to be a strong sign of aberrant/repressed ETC function^30–32^.

In addition, previous studies have revealed that FP not only targets fast proliferating cancer cells, it may also eliminate dormant-state cancer cells^33–35^. In order to confirm this and to explore the mechanism of FP induced prostate cancer cell death, we studied the sensitivity of FP treatment in slow-cycling DU145 cells. DU145^WT^ and DU145^FP^ cells were serum-deprived for 72 hours, and the cells were then seeded into plates, which contained medium with or without 10% fetal bovine serum (FBS) and 400 nM FP in different combinations. After 24 and 72 hours incubation, the cell viabilities were determined by using flow cytometry and Annexin V/7-AAD double staining assay. The cell numbers were counted by using an automatic cell counter before passing through the flow cytometer. As shown in Figure S4A, the DU145^WT^ cells cultured in FBS-free media (WT S − F−) turn into slow-cycling status in contrast to the DU145^WT^ cells cultured in the medium with 10% serum (WT S + F−).

Interestingly, the 400 nM FP treatments induce significantly more cell deaths in the slow-cycling DU145^WT^ cells, since after 24 and 72 hours of 400 nM FP treatments, the fast proliferating DU145^WT^ cells (WT S + F+) reveal significantly more viable cells than the DU145^WT^ cells in slow-cycling (WT S − F+) (Figure S4B and C). In contrary, the cell viabilities of the DU145^FP^ cells treated with 400 nM FP for 72 hours with and without serum in the medium are similar (~92.2% vs. ~89.9%; FP S + F+ vs. FP S − F+). To further explore the mechanisms involved in the sensitivity of FP in prostate cancer cells, we next investigated the effect of FP treatment in the DU145^FP^ cells with suppressed mitochondrial function.

### The FP induced DU145 cell apoptosis is related to mitochondrial function

Many previous studies have revealed that FP eliminates cancer cells by inducing apoptosis, which often requires well-maintained mitochondrial function^36, 37^. Our above results confirm that DU145^WT^ cells have relatively well-maintained mitochondrial function and are sensitive to FP treatment. Therefore, we next investigated how mitochondrial functional deficiency affects the FP induced apoptosis in DU145 cells. We compared the DU145^FP^ cells with our previously established mtDNA depleted DU145 cell model (DU145^MtDP^ line), which displays dysfunctional mitochondria and glycolysis dependent survival^29^. The DU145^FP^, DU145^MtDP^ and DU145^WT^ cells were treated with 400 nM FP for 24–72 hours, and apoptotic ratios were measured by flow cytometry (Annexin V-FITC/PI double staining). Since DU145^MtDP^ cells require extra pyruvate and uridine (PU) for survival, an equal amount of PU was added to all cell lines during the experiments. As shown in Fig. 2B in the left side, the DU145^FP^ cells survive the 400 nM FP treatment, and no significant apoptosis is observed at any point of time. The DU145^MtDP^ cells show a limited response to the FP treatment with a slight increase of apoptotic cells. However, the DU145^WT^ cells show a dramatic increase of late stage apoptotic cells comparing to the other cell types at the same point of time. The PU does not induce apoptosis in any cell line. Histograms and line charts in Fig. 2B in the right side show that 72 hours treatment has eradicated ~50% of the DU145^WT^ cells, whereas only ~15% of the DU145^MtDP^ cells were eradicated. Collectively, our data shows that the DU145^MtDP^ cells with dysfunctional mitochondria are more tolerated to FP treatment compared with the parental DU145^WT^ cells.

### The DU145^FP^ cells show up-regulation of anti-apoptotic genes

To further clarify the molecular basis of DU145^FP^ cells’ apoptosis resistance to FP treatment, we explored the expression status of a series of pivotal anti- and pro-apoptosis related genes in the DU145^FP^ cells by performing transcriptome analysis. As shown in Fig. 2C, the anti-apoptotic genes *BCL2*, *BID*, *BAG3* and *BAG1* are all found up-regulated in the DU145^FP^ cells (~2.3, ~1.7, ~1.3 and ~1.3 folds, respectively, DU145^FP^ vs. DU145^WT^ in Log_2_ transformed ratio of FPKM values, FP/WT). However, we found that three of the four inhibitors of apoptosis genes (IAP) were also up-regulated, including *BIRC3*, *BIRC2* and *BIRC5* (*SURVIVIN*) (~3.5, 2.5 and 1.1 folds, respectively, DU145^FP^ vs. DU145^WT^). A more detailed gene expression profile is shown in Figure S5. Although the expression of pro-apoptotic genes *BOK* and *BAD* were up-regulated (2.0 and 1.2 folds, respectively, DU145^FP^ vs. DU145^WT^), it turned out that the antisense mRNA of *BOK* (*BOK-AS1*) was simultaneously up-regulated (~7.8 folds, DU145^FP^ vs. DU145^WT^) as well. As shown in Fig. 2D, the immunoblotting results reveal that the DU145^FP^ cells contain significantly increased protein products of BCL-2 and BID, slightly increased BAG3, BAD and BIRC5 (SURVIVIN), and relatively stable levels of BAK, XIAP, and CYCS (Cytochrome C), which are all consistent with the results of the transcriptome analyses. However, the protein products of BOK and CASP9 (CASPASE 9) show inconsistencies with their mRNA expression levels.

Overall our analysis shows that genes contributing to cell survival signaling pathways seem to be universally up-regulated, while the genes directly participating in the apoptotic process are generally down-regulated. The detailed analysis and visualized molecular pathways is shown in Figure S6. Collectively, these results suggest that the DU145^FP^ cells contain overall higher level of anti-apoptotic proteins, which might directly contribute to the FP resistance.

### The FP treatment initiates metabolism reprogramming in the DU145 cells

Our data indicates that the DU145^FP^ cells are under continuous mitochondrial metabolic stress, which might facilitate development of alternative metabolism preferences over OXPHOS to support the cells’ survival.

**Figure 3:**
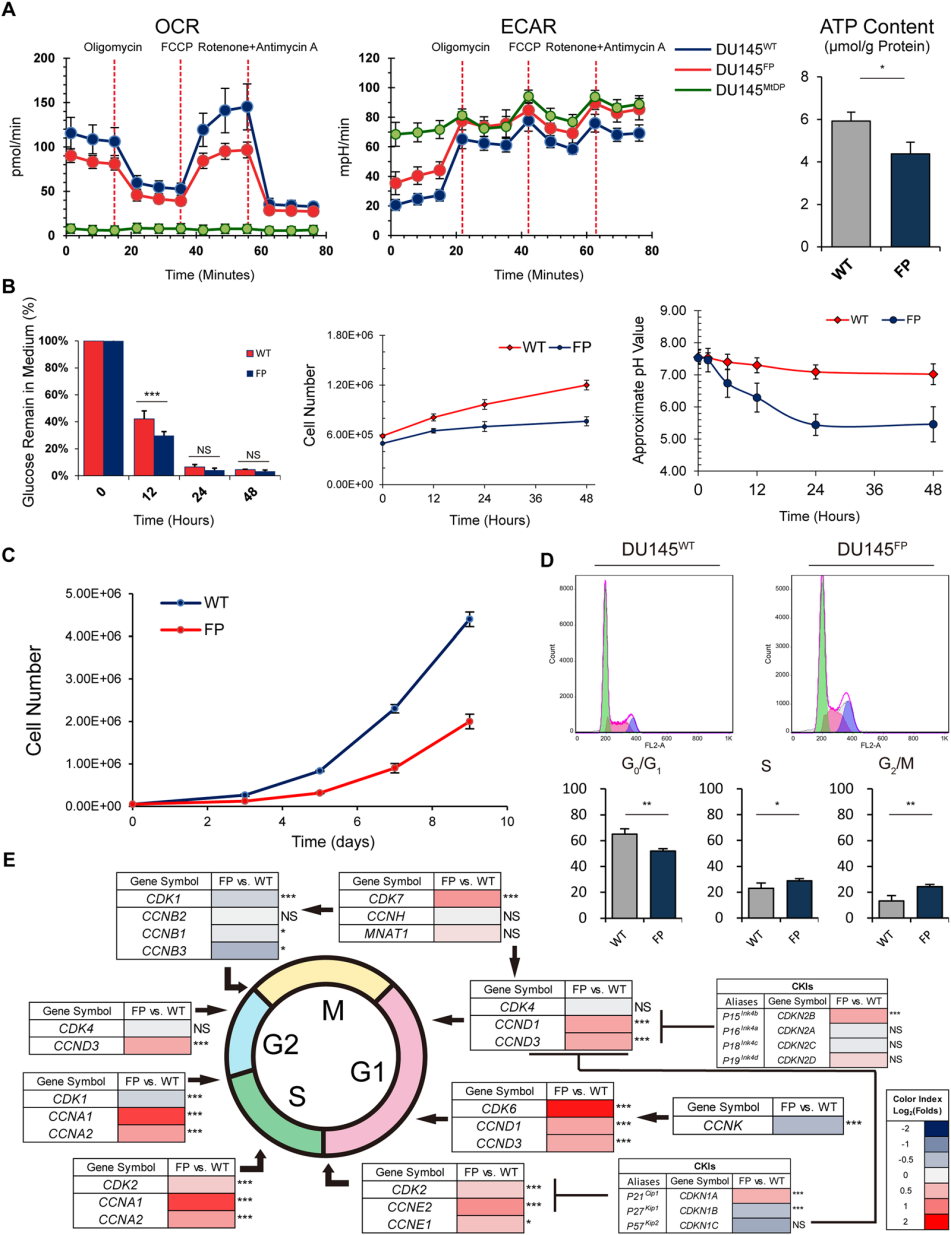
DU145^FP^ cells show reprogrammed metabolism and are slow cycling. (**A**) The Seahorse XF Cell Mito Stress assay were applied to show cells’ mitochondrial respiration capacity and metabolic preference. The intracellular ATP concentrations are measured based on bioluminescence assays. The data is presented as means from three independent experiments, each experiment includes at least three parallel samples (mean ± S.D). (**B**) The glucose consumption status at 12, 24 and 48 hours are shown in line charts, and corresponding cell counting results are presented by line charts in the middle. For the long-term extracellular acidification analysis, all cells were maintained in non-buffered Seahorse medium for 12, 24 and 48 hours. Then the approximate pH values of cell culture supernatants were measured and shown in the line charts on the right. The data is presented as means from three independent experiments, each experiment includes two parallel samples (mean ± S.D). (**C**) Cell growth curves obtained from the MTT assay in a period of 0–11 days. After 11 days of cultivation, the relative cell density is estimated by the absolute OD value. The data is presented as means from three independent experiments (mean ± S.D). (**D**) The cell cycle is analyzed by PI staining by flow cytometry. A representative group of analyses is shown in the histograms, and the Watson’s model is applied to separate the different cell phases. The data is presented as means from five independent experiments (mean ± S.D). (**E**) Transcriptome analyses showing up- or down-regulation of cell cycle-related molecules including CDKs, CKIs and cyclin members. The color-coded bars index Log_2_ transformed ratio of FPKM values (e.g., FP/WT). Statistical significance: *p < 0.05, **p < 0.01, ***p < 0.001.

To test this possibility, we firstly assessed the cells’ oxygen consumption rate (OCR) and extracellular acidification rate (ECAR) by using a Seahorse extracellular flux analyzer with the Mito Stress Test. The DU145^MtDP^ cells were included as a reference control. As shown in Fig. 3A in the left side, the OCR values of the DU145^FP^ cells are lower than that of the DU145^WT^ cells, whereas the ECAR values are consistently higher than that in the DU145^WT^ cells. At resting state (basal line), the DU145^FP^ cells have ~1.3 folds lower OCR but ~1.7 folds higher ECAR than the DU145^WT^ cells. Furthermore, the maximum respiratory potential of the DU145^FP^ cells is significantly weaker than that in the DU145^WT^ cells, and the ATP production capacity of the DU145^FP^ cells is also lower (see the instruction of the Seahorse Mito Stress Test). As the reference, the DU145^MtDP^ cells exhibit minimal OCR (~15 folds lower than DU145^WT^ cells), but a maximum ECAR (~3.5 folds higher than DU145^WT^ cells). To verify the ATP production capacity, we measured the intracellular ATP concentrations in the DU145^FP^ and DU145^WT^ cells by using a commercial ATP-test kit based on luciferase activity. As shown in Fig. 3A in the right side, the ATP concentration in the DU145^FP^ cells is ~1.35 folds lower than that in the DU145^WT^ cells, which is generally consistent with the Seahorse Mito Stress Tests (OCR) shown in the Fig. 3A in the left side.

It is known that the decreased OCR and increased ECAR of cancer cells are associated with a well-known metabolism peculiarity -the Warburg effect, which is usually accompanied by a sharply increased glucose consumption and enhanced extracellular acidification rate. Therefore, we next measured the glucose consumption ratio of the DU145^FP^ and DU145^WT^ cells. As shown in Fig. 3B in the left panel, the glucose consumption process of the DU145^FP^ cells is significantly faster than that of the DU145^WT^ cells (~15% more glucose consumption in the DU145^FP^ cells after 12 hours). However, the cell proliferation rate of the DU145^FP^ cells is significantly slower than that in the DU145^WT^ cells (Fig. 3B, middle panel). To investigate the extracellular acidification rate, the DU145^FP^ and DU145^WT^ cells were maintained in Seahorse non-buffered DMEM medium for 0–48 hours. The pH values of cell culture supernatants were determined by using Seahorse sensor cartridges with an inverted fluorescence microscope. As shown in Fig. 3B in the right panel, the pH value of the DU145^FP^ cells’ culture medium descends fast from ~pH 7.5 to ~pH 5.4 within the first 24 hours. However, the corresponding DU145^WT^ cells’ medium pH fluctuation only shows a reduction in a range from ~pH 7.5 to pH 7.09. The related fluorescence images and cell counting results are shown in the Supplemental Figure S7A. Collectively, these results reveal that the DU145^FP^ cells exhibit a significantly stronger glucose consumption capability as well as extracellular acidification ratio than the DU145^WT^ cells, all illustrating a stronger aerobic glycolysis as a reprogrammed cell metabolism status.

### DU145^FP^ cells are slow cycling and show prolonged G2/M cell phase

Increasing evidence suggests that cancer stem-like cells often are dormant with a prolonged cell cycle^38^. We next monitored the proliferation kinetics of the DU145^FP^ cells, while their cell cycle distributions were assessed with flow cytometry. As shown in Fig. 3C, the DU145^WT^ cells show a fast proliferation manner. In comparison, the growth curve of the DU145^FP^ cells is comparatively flatter. At day 9, the DU145^WT^ cells show ~2.2 folds larger cell population than the DU145^FP^ cells.

To study the cell cycle, we fixed the cells with formalin, treated them with sufficient RNase A and finally stained them with propidium iodide (PI). As shown in Fig. 3D, the DU145^FP^ cells show a significantly reduced G_0_/G_1_ phase proportion (~65% vs. ~52%, DU145^WT^ vs. DU145^FP^), but the S and G_2_/M phase percentages are significantly larger (~23% vs. ~29% and 13% vs. 24%, respectively, DU145^WT^ vs. DU145^FP^).

To further explore the cell cycle arrest phenomenon, we performed transcriptome analyses and focused on the cell cycle machinery. As shown in Fig. 3E, the genes contributing to the most of cell cycle phases have been universally up-regulated in the DU145^FP^ cells. However, only the expressions of the G2/M transition required genes have been down-regulated. A complete expression profile is shown in Figure S7B. The top three up-regulated CDK family members are *CDK6*, *CDK19* and *CDK5* (1.8, 1.3 and 0.8 folds, respectively, DU145^FP^ vs. DU145^WT^). The top three down-regulated CDK family members are *CDK18*, *CDK9* and *CDK17* (−1.2, −0.5 and −0.3 folds, respectively, DU145^FP^ vs. DU145^WT^). The top three up-regulated cyclin family members are *CCNA1* (*CYCLIN A1*), *E2* and *A2* (1.5, 0.8 and 0.7 folds, respectively, DU145^FP^ vs. DU145^WT^). The top three down-regulated cyclin family members are *CCNF*, *B3* and *K* (−0.8, −0.6 and −0.5 folds, respectively, DU145^FP^ vs. DU145^WT^). For the CDK inhibitors (CKIs), only the *CDKN1B* (*P27* ^*KIP1*^) gene expression is down-regulated, whereas the remaining members such as *CDKN2B* (*P15* ^*INK4B*^), *CDKN1A* (*P21* ^*CIP1*^) and *CDKN3* (*CIP2*) are all up-regulated according to the gene expression analyses (0.6, 0.6 and 0.4 folds, respectively, DU145^FP^ vs. DU145^WT^). These data therefore suggests that direct inhibition of CDKs by FP treatment might not be the solely mechanism responsible for the slow cycling feature of the DU145^FP^ cells, whereas more extended remodeling of the cell cycle machinery might contribute to this as well.

### DU145^FP^ cells show significantly higher migratory potential

**Figure 4:**
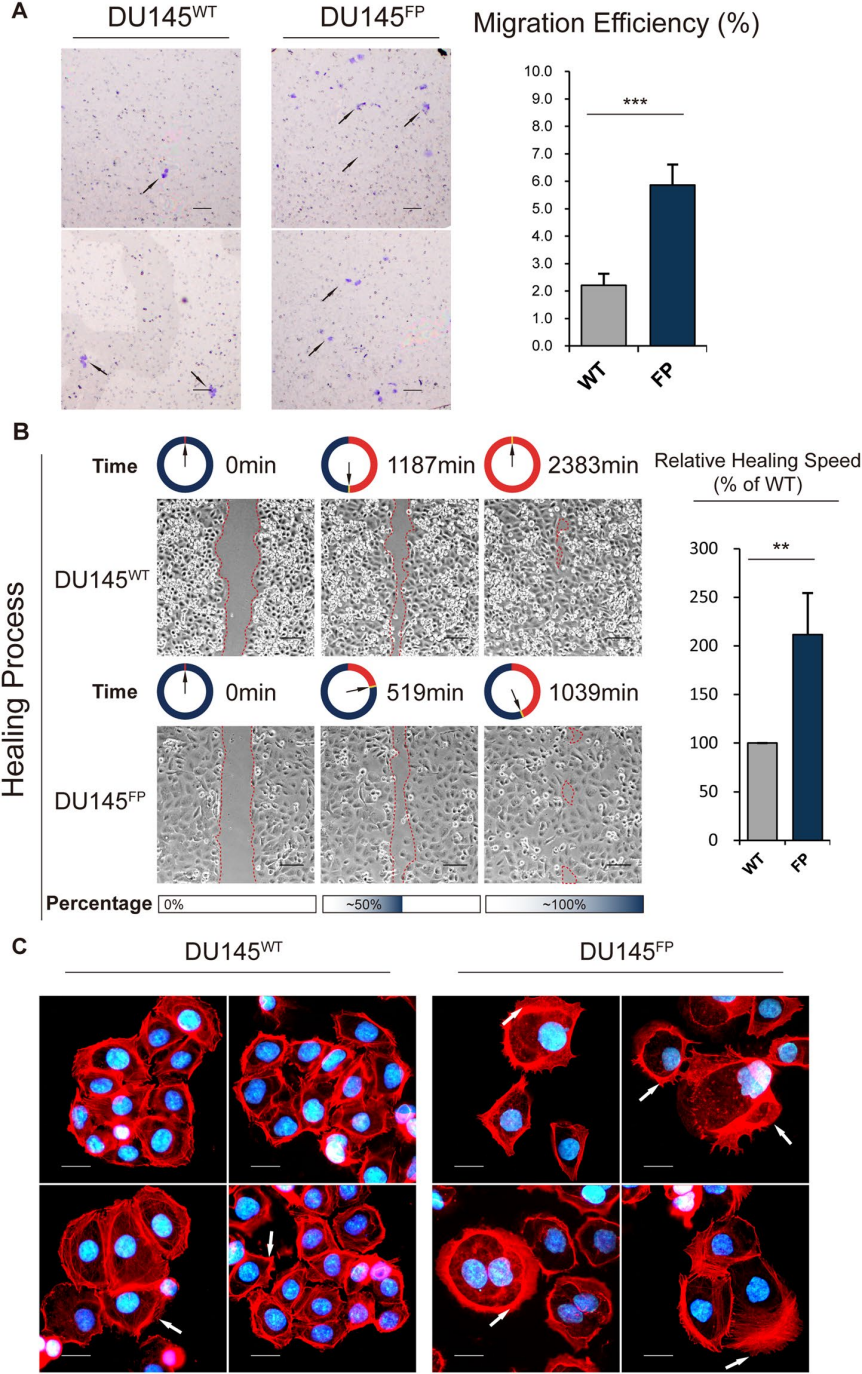
DU145^FP^ cells display a higher mobility and well-developed lamellipodia. (**A**) Cell motility assessed by transwell assays. The data is presented as means from three independent experiments, each experiment includes at least two parallel samples (mean ± S.D). The bars are equal to 200 µm. (**B**) The wound-healing assay obtained by live cell imaging system. The red dashed lines indicate the scratch area. The wound widths and corresponding relapsed time were measured using software Adobe Photoshop and Microsoft Excel, and the cells’ healing speeds were calculated. The data is presented as means from three independent experiments (mean ± S.D). The bars are equal to 100 µm. (**C**) The cells were stained with the rhodamine conjugated phalloidin for the purpose of visualizing F-actin and lamellipodia. The cell nuclei were co-stained with DAPI showing in blue. The bars are equal to 20 µm. Statistical significance: *p < 0.05, **p < 0.01, ***p < 0.001.

During our time-lapse video experiments, we observed that the DU145^FP^ cells displayed greater motility and EMT-like phenotype features, which are associated with cancer metastasis and cancer stem cells^39, 40^. We therefore further investigated the motility of the DU145^FP^ and DU145^WT^ cells. As shown in Fig. 4A in the left side, more DU145^FP^ cells are found penetrated through the 8 µm diameter pore trans-well membranes comparing to their parental wild type cells. The quantification results represented in the histograms in Fig. 4A in the right side show significantly greater migration abilities in the DU145^FP^ compared with the DU145^WT^ cells (~6% in the DU145^FP^ vs. ~2% in the DU145^WT^). We also performed wound healing assays, by using a live-cell imaging system that recoding time-lapse video, and the cells’ healing speed was calculated (Video SV3). Representative results, including images and histograms, are shown in Fig. 4B. The red dashed lines indicate the wound area. We found that the DU145^FP^ cells recover ~50% of the wound area after ~519 minutes, whereas the DU145^WT^ cells require ~1187 minutes to do the same. Simplified, about ~1000 minutes were required for the DU145^FP^ cells to complete the healing process, whereas approximately 2000 minutes were required for the DU145^WT^ cells. The healing speed of the DU145^FP^ cells is thus about twice as fast than the one of the DU145^WT^ cells. It should be noted that although the DU145^FP^ cells healed the wound in a shorter time, these cells were always sparsely distributed in the plates, whereas the DU145^WT^ cells grew faster but with higher cell density.

Enlarged cell sizes were observed in the DU145^FP^ population, frequently shown as atypical giant cells, indicating well-developed lamellipodia. Many studies have revealed that lamellipodia plays pivotal roles in the cells migration and invasion processes^41–43^. Hence, we stained the DU145^FP^ and DU145^WT^ cells with rhodamine-conjugated phalloidin to visualize F-actin, which is the main constituent of lamellipodia^44, 45^. As shown in Fig. 4C, the DU145^FP^ cells display strong staining of rhodamine-conjugated phalloidin, which is indicated by the high-density red color and is pointed with white arrows. In addition, more filopodia have also been observed in the DU145^FP^ cells. In contrast, the DU145^WT^ cells are morphologically smaller and possess less lamellipodia and filopodia development. Collectively, these results suggest that the DU145^FP^ cells are highly migratory and thereby might have a higher metastatic potential.

### The DU145^FP^ cells exhibit decreased apoptotic sensitivity to cisplatin and docetaxel treatment

Platinum and taxane drugs are commonly used chemotherapeutic agents to treat cancer patients^46–48^. We next investigated the apoptotic response of the DU145^FP^ cells to additional cisplatin and docetaxel treatments. The drug concentrations were chosen based on previous publications: 30 µM and 60 µM were used for cisplatin, and 10 nM and 20 nM for docetaxel respectively in our experiments^49, 50^. The control cells were treated with equal amounts of solvent (DMSO). The viable cells were determined by using flow cytometry, based on the Annexin V-FITC/PI double staining assay. Representative results are shown in Figure S8. The cells identified as Annexin V-FITC/PI double negative phenotype are considered viable, whereas the Annexin V-FITC/PI double positive phenotype is considered as late stage apoptosis.

**Figure 5:**
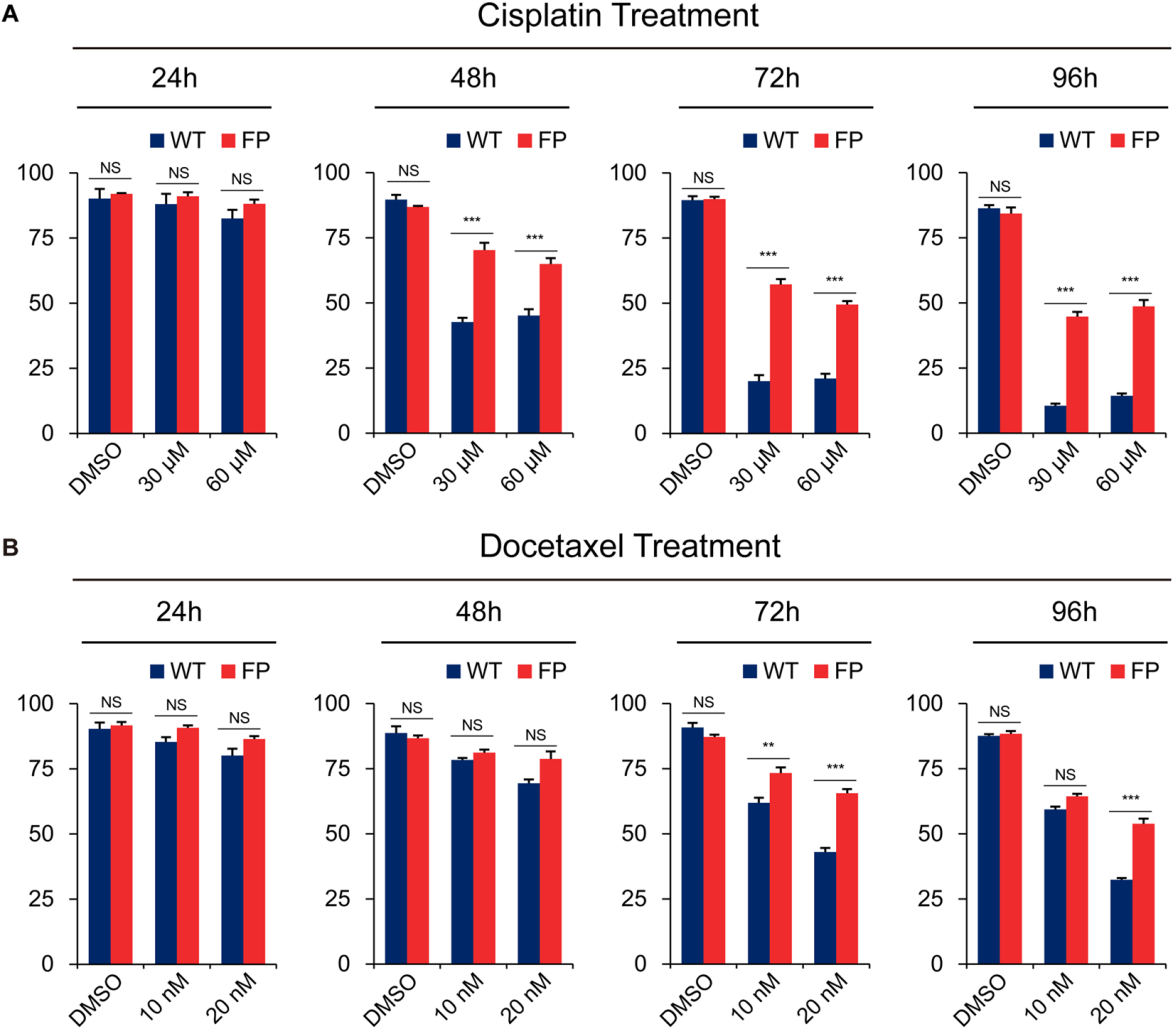
DU145^FP^ cells are less sensitive to cisplatin and docetaxel-induced apoptotic pressure. (**A**) The cisplatin and (**B**) docetaxel treatments were applied to the cells for 24–96 hours to evaluate the efficacy of apoptosis induction. The subpopulations’ double negative phenotype for Annexin V-FITC and PI staining were considered as viable (see Supplementary Material). The numbers of viable cells were collected, and displayed in the histograms. The data is presented as means from four independent experiments (mean ± SEMs. one-way ANOVA analysis was applied with Bonferroni method). Statistical significance: *p < 0.05, **p < 0.01, ***p < 0.001.

For each treatment at 24–96 hours, the proportions of viable cells are summarized and presented in the histograms (Fig. 5A and B). In short, for both drugs at any concentration, the DU145^FP^ cells always exhibit more viable than the DU145^WT^. Regarding the cisplatin treatments, a stronger time-dependent effect is observed in both the DU145^WT^ and DU145^FP^ cells, but the concentration-dependent effect is relatively weak in the DU145^WT^ cells. However, both time-dependent and concentration-dependent effects are significantly stronger regarding the docetaxel treatments in the DU145^FP^ and DU145^WT^ cells. To conclude, after 96 hours of treatment, 30 µM and 60 µM cisplatin applications eliminated ~50% and ~55% of the DU145^FP^ cells, respectively, whereas ~86% and ~91% of the DU145^WT^ cells were killed. For docetaxel, 96 hours of 10 nM and 20 nM treatment resulted in ~36% and ~46% DU145^FP^ cells death, while ~40.6% and ~67.7% of the DU145^WT^ cells were eliminated, respectively. Taken together, these data suggests that the DU145^FP^ cells are less sensitive to the apoptotic pressures exerted by both cisplatin and docetaxel treatments.

### The DU145^FP^ cells overexpress a series of ABC-family members

Some studies have suggested that overexpression of ABCG2 is associated with enhanced FP treatment resistance in some certain cell types^51–53^, since the overexpressed ABCG2 transporter can facilitate the efflux of FP molecules from the cytoplasm. Moreover, the overexpression of many other ABC-family members are also correlated with enhanced chemotherapeutic resistances^54–56^. To determine this hypothesis, we next assessed the expression of ABCG2 and other ABC-family members.

**Figure 6:**
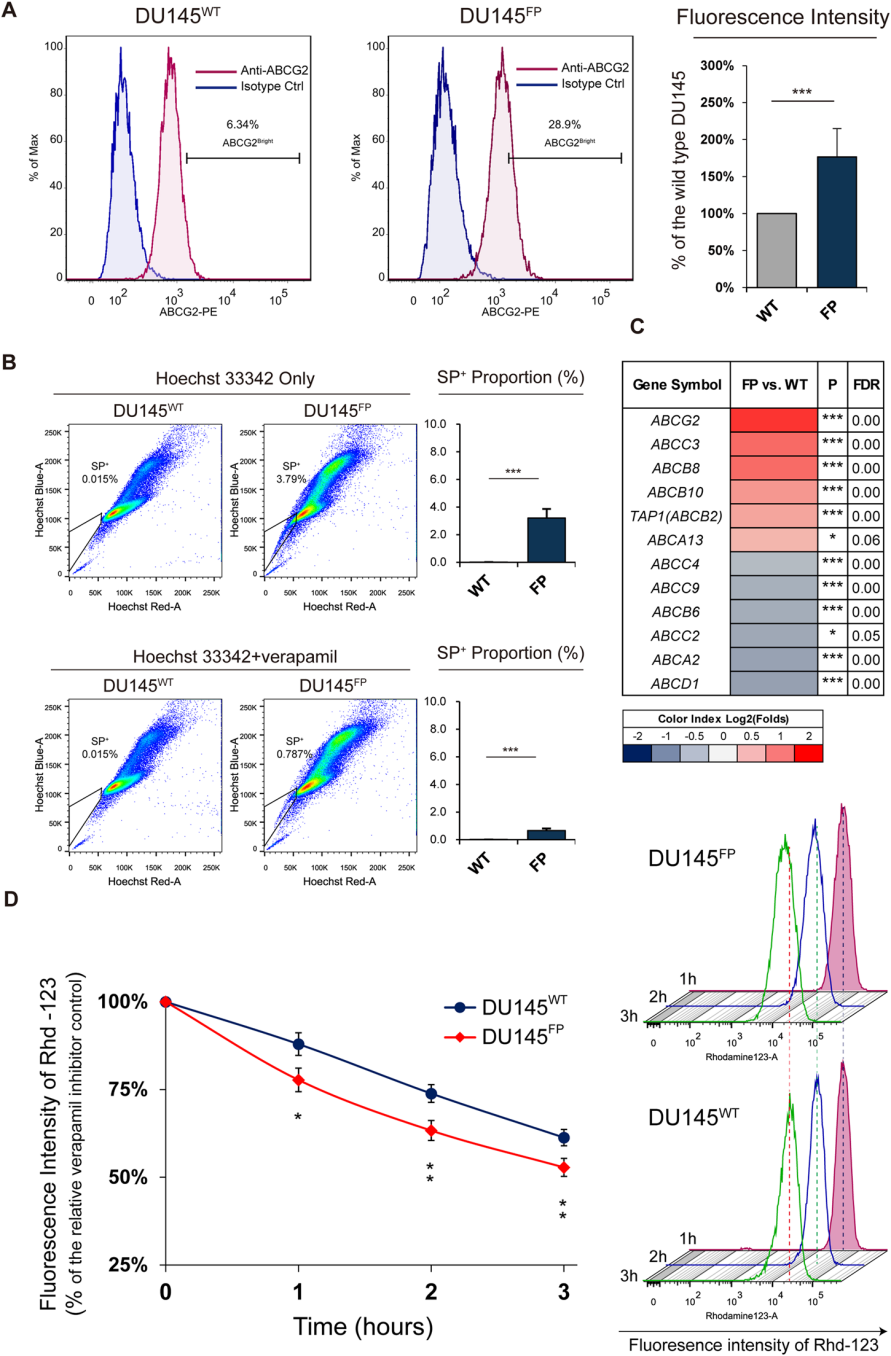
DU145^FP^ cells express higher levels of several ABC family members. (**A**) Cells were stained with phycoerythrin (PE) conjugated monoclonal antibody against ABCG2 by flow cytometry. The geometric mean values of the full fluorescence area were applied for further calculations. Summarized results are shown in the histograms on the right. The data is presented as means from three independent experiments (mean ± S.D). (**B**) Side population cells (SP + ) were detected by using Hoechst 33342 staining and flow cytometry. The SP + cells are displayed as the tail-like subpopulations in the frame on the left. Sufficient ABCG2 inhibitor verapamil is given for control staining. The data is presented as means from three independent experiments (mean ± S.D). (**C**) Transcriptome analysis of ABC-family members. The six most up- and down-regulated members are listed in the heat map. The Log_2_ transformed ratio of FPKM values (e.g., FP/WT) are indicated by color-coded index bars. (**D**) The Rhd-123 efflux capacities. Shown on the right, the dashed lines are normalized to the approximate center of the fluorescence area of wild type cells to contrast the reduced fluorescence intensity in FP resistant cells. On the left, the line chart reflexes the extent of the reduced Rhd-123 concentration in both cell lines from the Rhd-123 exclusion assay. The data is presented as means from four independent experiments (mean ± S.D). Statistical significance: *p < 0.05, **p < 0.01, ***p < 0.001.

As shown in Fig. 6A, we confirm that the average ABCG2 expression level of the DU145^FP^ cells are about 1.76 folds higher than that of the DU145^WT^ cells (~176% vs. 100%, DU145^FP^ vs. DU145^WT^), revealed by flow cytometry with fluorophore conjugated monoclonal antibody.

Furthermore, it is known that the ABCG2 overexpression is associated with the side population phenomena (SP^+^)^57^, therefore we next performed side population assays to verify the current results. As shown in Fig. 6B, the SP^+^ cells are identified as a tail-like cell sub-population in the gates close to the G_1_/G_0_ cell clusters. According to the data, we found ~3.79% SP+ cells in the total DU145^FP^ cell population, but no typical SP^+^ cells were detected in the DU145^WT^ cells.

The ABC-family members are known for playing various roles in chemicals’ exclusion and thus contributing to chemotherapy resistance. We therefore summarized the top six up-regulated and top six down-regulated ABC-family members in a heat map based on our transcriptome analyses. As shown in Fig. 6C, the most up-regulated ABC family members are *ABCG2*, *ABCC3* and *ABCB8* (1.6, 1.1 and 1.1 folds, respectively, DU145^FP^ vs. DU145^WT^), whereas the most down-regulated members are *ABCD1*, *ABCA2* and *ABCC2* (−0.7, −0.7 and −0.7 folds, respectively, DU145^FP^ vs. DU145^WT^). The transcriptome analyses confirm the overexpressed ABCG2 in the DU145^FP^ cells.

To further evaluate the potential contributions of the ABC-family members for drug efflux in the DU145^FP^ cells, we performed rhodamine 123 (Rhd-123) exclusion assay. As shown in Fig. 6D in the right side, fluorescence intensities of Rhd-123 in the DU145^FP^ cells are always lower than that in the DU145^WT^ cells after 3 and 4 hours incubation at 37 °C. The respective results for inhibitor controls is shown in Figure S9. Furthermore, we also normalized the fluorescence intensity of Rhd-123 detected in the DU145^WT^ and DU145^FP^ cells to their respective inhibitor controls, which represent the maximum potential of the cells to load Rhd-123 probe. The results are shown in the line-chart in Fig. 6D in left side. The results indicate that the DU145^FP^ cells exhibit overall ~10% stronger capacity to exclude the intracellular Rhd-123 probes than their parental DU145^WT^ cells.

### DU145^FP^ cells overexpress a series of CSC-associated molecular markers

**Figure 7:**
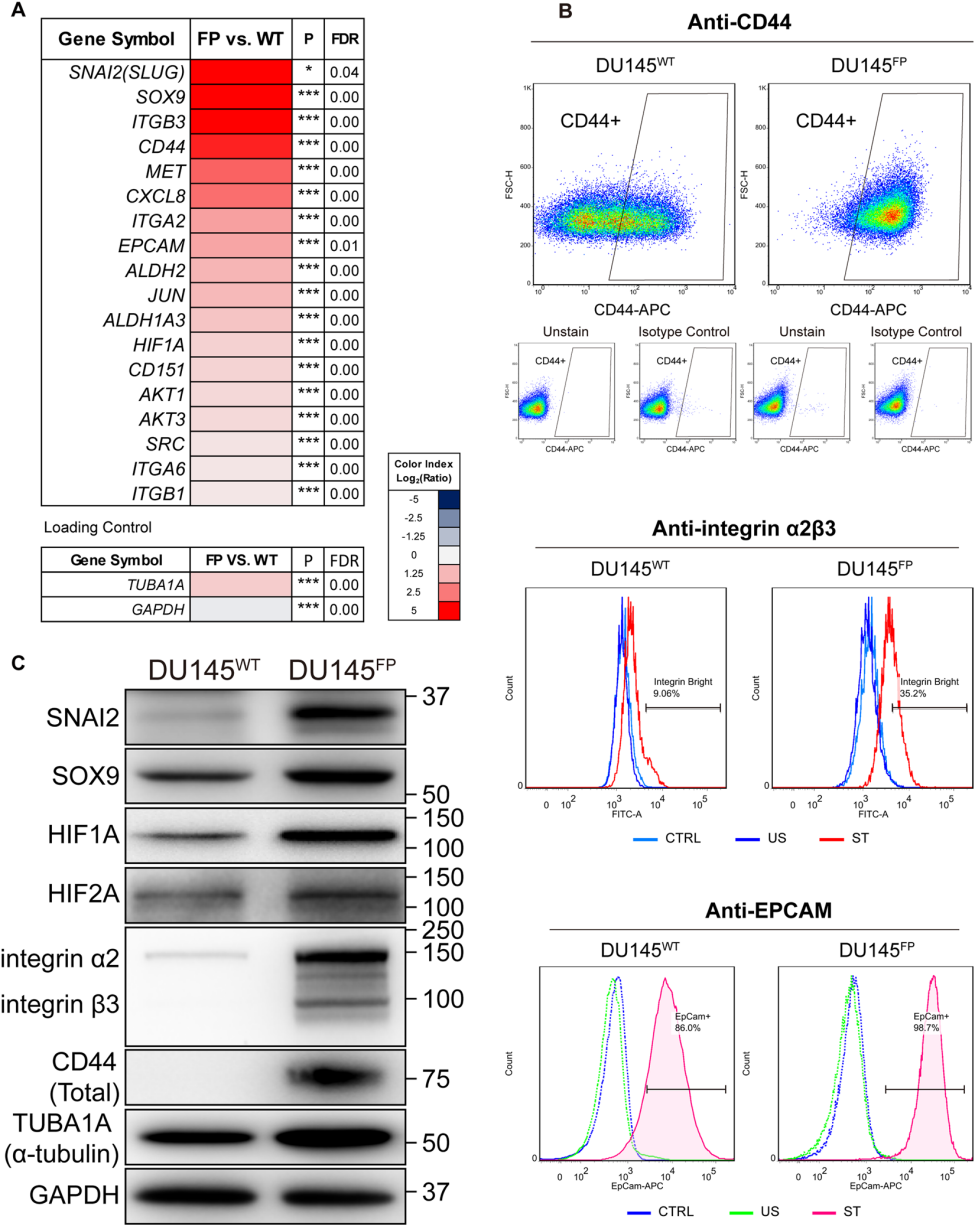
The DU145^FP^ cells express higher levels of cancer stemness related genes. (**A**) Transcriptome analyses of prostate cancer stemness-related markers. The Log_2_ transformed ratio of FPKM values (e.g., FP/WT) are indicated by color-coded index bars. (**B**) Cells were stained with fluorophore conjugated monoclonal antibodies against CD44 and EPCAM, the antibody against integrin α2β3 without fluorophore conjugation was detected by using secondary antibody conjugated with fluorophore, and stained cells were analyzed by flow cytometry. The scatter plots and histograms show representative results. CTRL denote cell stained with isotype control antibody or only secondary antibody; ST denote cell stained with antibody against EPCAM and integrin α2β3; US denote unstained cells. (**C**) Immunoblotting confirmation of the protein expression of A-tubulin, GAPDH, SOX9, SLUG, HIF1A, HIF2A, CD44 and integrin α2β3.

Besides the significantly higher level of ABCG2 expression and enlarged SP^+^ subpopulation cells, the DU145^FP^ cells overexpress a series of other CSC-related markers as well. As shown in Fig. 7A, the top five up-regulated cancer stemness associated genes include: *SNAI2* (*SLUG*) (8.2 folds), *SOX9* (6.4 folds), *ITGB3* (*integrin β3*) (5.4 folds), *CD44* (4.3 folds) and *MET* (2.9 folds) (DU145^FP^ vs. DU145^WT^). In addition to these, other cancer stemness associated genes including *EPCAM* (1.6 folds), *ALDH2* (1.3 folds), *ALDH1A3* (0.9 folds) and *HIF1A* (0.7 folds) are all found up-regulated though to less extent. We verified a series of these CSC-associated markers by immunoblotting and flow cytometry. Representative results are shown in Fig. 7B and C. These observations are all in good agreement with our transcriptome analyses, revealing that the DU145^FP^ cells are highly CD44 positive (~93% in DU145^FP^ vs. ~45% in DU145^WT^). The DU145^FP^ cells also display increased integrin α2β3 (~30% in DU145^FP^ vs. ~9% in DU145^WT^) and EPCAM (~98% in DU145^FP^ vs. ~83% in DU145^WT^). The immunoblottings demonstrate that the protein products of SOX9, SNAI2, HIF1A, CD44 and integrin α2 and β3 are increased in the DU145^FP^ cells, which are all in line with the transcriptome analyses and flow cytometry.

## Discussion

In recent decades, kinase inhibitors, including CDK inhibitors, have shown a great potential in targeted therapies^14, 15, 58^. FP is a pan-CDK inhibitor, which is efficacious in eliminating both proliferative and dormant cancer cells^24, 33, 35^. Unfortunately, the clinical trials were disappointing as various aggressive cancers exhibited extensive resistance to FP treatment^59^. Although some studies have suggested that FP resistance can be directly associated with the up-regulation of cancer stemness features, e.g. the overexpression of ABCG2^53, 60^, the precise molecular mechanism remains unclear.

In this study, we have shown that FP treatment induces intense apoptosis of DU145 cells, which is dependent on well-maintained mitochondrial function. To our best knowledge, we have reported for the first time that the FP treatment induced mitochondrial lesions also stimulate metabolism reprogramming in the DU145 cells, and the survived cells become more dormant and aerobic glycolysis addictive. Furthermore, our study suggests that this FP induced metabolic reprograming provides cancer cells with stronger survival capabilities in noxious microenvironment, e.g. attacking by additional therapeutic drugs. Moreover, FP resistant cells show aggressive neoplastic features and increased expression of stem cell related proteins, which are all associated with cancer cell stemness.

The FP treatment has been shown to induce apoptosis in various cancer cells, such as T cell leukemia, B cell leukemia, breast-, gastric- and NSC lung cancer^24^. Previous studies suggested that FP induced cell apoptosis should be via multiple p53 independent pathways^24, 61, 62^. Our current study supports this conclusion, because the p53-null human prostate cancer PC3 cell line was also proven sensitive to 400 nM FP treatment, as revealed for the LNCaP and DU145 cells (Figures S10 and 1). Furthermore, it has been shown that FP treatment results in mitochondrial depolarization in diverse cancer cell lines such as the SW2 and H69, and that mitochondrial depolarization activates caspase 3 leading to apoptosis^25, 63^, confirming the involvement of mitochondrial function in the FP mediated apoptosis.

Many studies have demonstrated that FP down-regulates the expression of a series of anti-apoptotic genes such as *BCL2*, *BCL-xL*, *MCL-1*, *XIAP* and *BIRC5*, with the consequence of increased cell apoptosis^64–67^. Indeed, the DU145^FP^ cells exhibit a universally up-regulated expression of these genes such as *BCL2*, *BID* and *BAG3*. While the previous studies are based mainly on the short-term FP treatments, typically within 3 to 72 hours, our DU145^FP^ cells are allowed for a long period of adaptation and gradual gene expression adjustment. Hence, the reduced expressions of the anti-apoptotic genes in short-term FP treated cells should be reasonable with those dramatically increased apoptotic cells. Therefore, our results suggest that the DU145^FP^ cells have developed a necessary molecular basis, including the expression of more anti-apoptotic proteins to compensate the pro-apoptotic pressure produced by the 400 nM FP treatment. In support of our results, other studies also reported the up-regulation of anti-apoptotic genes after FP treatment^25, 68^. Collectively, our data therefore indicates that the regulation of anti-apoptotic machinery in FP treated cells may be a time-dependent process involving multiple molecular mechanisms.

Studies have shown that 400 nM FP is sufficient to inhibit most of the CDKs’ activities^24^. However, the DU145^FP^ cells keep dividing, although slowly. It should be noted that the surviving DU145^FP^ cells experienced a dormant period during the adaptation process. Our cell cycle-related gene analyses were performed only after the adaptation process was successfully accomplished, therefore the DU145^FP^ cells have most likely established a new gene expression profile compared with the cells during the quiescent adaptation process. Unfortunately, we did not perform gene expression profiles for all of the cells in different phases during that process since the surviving cells are too few to collect. Nevertheless, our gene expression profile of the DU145^FP^ cells reveals complex cell cycle machinery remodeling. The CDK and cyclin members required for the G2/M phase transition are significantly down-regulated, but the expression of genes involved in the remaining cell cycle phases or check points are universally up-regulated. These results are in line with the G2/M cell phase accumulation of the cells seen by the flow cytometry, suggesting that the slow cycling and slow progression of the DU145^FP^ cells through the G2/M phase is not only due to the direct FP inhibition on CDKs. In addition, it is known that FP is a substrate of the transporter ABCG2^51, 53^. Theoretically, the overexpression of ABCG2 in the DU145^FP^ cells can result in decreased intracellular FP concentration, which might be partly supported by the results of Rhd-123 efflux assay. Therefore, our results suggest that there are multiple factors contributing to the slow cycling feature of the DU145^FP^ cells, which at least include epigenetic remodeling, direct pharmacological cell cycle inhibition (suppressed CDK activity) and a possible reduction of the intracellular FP concentrations.

Multiple previous studies have shown that exposure to FP induces significant mitochondrial lesions in diverse cancer cell lines, and that direct mitochondrial damage can be revealed by the elevated frequency of mitochondria depolarization^25, 26, 63, 69, 70^. The DU145^FP^ cells experienced a uniquely long adaptation process to FP treatment showing mitochondrial function alterations. During this process, less than 1% cells survived, suggesting that those few cells should possess greater plasticity of switching their metabolic framework. Indeed, we have confirmed that the DU145^FP^ cells have a stronger metabolic preference towards anaerobic glycolysis (lower OCR and higher ECAR under normoxic circumstance) with lower ATP production, which is consistent with the enhanced glucose uptake, faster extracellular acidification and the increased mitochondrial depolarization events. These observations indicate that, besides the apoptotic effect, the mitochondrial damages induced by the FP treatment can also initiate metabolism reprogramming in the DU145 cells. Mitochondrial dysfunction is often proven linked to advanced neoplastic features^71–73^, and assumed connect to cell metabolic reprogramming and cancer cell stemness up-regulation^74–77^, which are all verified in our current study as well.

In earlier studies, it has been demonstrated that FP may activate the ATPase activity of ABCC1 (MRP1)^60^, which directly contributes to chemotherapeutic resistance in diverse cancers^55, 78^. However, no studies have demonstrated that FP can directly affect ABCG2 expression. Hence, the overexpressed ABCG2 in the DU145^FP^ cells should be a consequence of adaptation process and might possibly be due to the restructured transcriptome. Furthermore, the DU145^FP^ cells not only show less sensitive to the apoptotic pressure exerted by additional therapeutic drugs, but also reveal a stronger efflux capacity of Rhd-123. However, there are studies suggested that ABCG2 does not respond to the efflux of taxane-based drugs and cisplatin^55^. This may indicate that other ABC-family members might also be involved in the reduced apoptotic sensitivity of the DU145^FP^ cells.

Besides the chemotherapy resistance, cancer metastasis is a main cause of mortality in patients and is usually associated with an aggressive cancer stem cell phenotype^79, 80^. Our *in vitro* assays show that the DU145^FP^ cells have acquired a higher migratory potential than the DU145^WT^ cells. One possible explanation is that the DU145^FP^ cells hold well-developed lamellipodia. It is known that the highly developed lamellipodia are indicators of stronger cell invasiveness, and are associated with the degradation of extracellular matrix (ECM) and thus mediate cancer metastasis^41–43^.

To investigate that multiple up-regulated cancer stemness factors in the DU145^FP^ cells, we observed a pivotal cancer stemness related signaling network, which is activated in the DU145^FP^ cells. This may possibly be mediated by the hypoxia-induced factors HIF1A and HIF2A, and is initiated by the FP-induced mitochondrial damages. The HIF-family members are transcription factors regulating a series of cell stemness related signaling pathways in both CSCs and normal stem cells (SCs)^81–85^. Interestingly, we have found that the most up-regulated cancer stemness related genes in the DU145^FP^ cells are those regulated by the HIF-family members^86–89^. Hence, our studies suggest that the hypoxia-induced signaling might be an up-stream driving event which enhances cell stemness during the development of FP resistance. In earlier studies, it is known that mitochondrial lesions can often disrupt the integration of mitochondrial electron transport chain, which in turn generates abundant ROS products^90^ that stabilize HIF1A protein^91^. In support to our above hypothesis, we found a series of mtDNA-encoded genes, as well as multiple ETC components down-regulated in the DU145^FP^ cells, which were correspondingly associated with the elevated ROS levels inside the DU145^FP^ cells. These experimental results therefore suggest that the FP-induced stemness phenotype may partially relate to the damages and functional alterations of mitochondrial homeostasis. Of note, the FP treatment might activate some other unknown signals that mediate cells’ responses to hypoxic environment, and facilitate the development of greater cancer stemness. Besides that, previous studies have shown that the co-expression of genes *Snai2* (*Slug*) and *Sox9* regulate the mammary stem cell (MaSC) status in mice^92^. Furthermore, SNAI2 (SLUG) is known to regulate EMT process^93, 94^, and the cells that undergo EMT process are often found to behave stem cell like properties. Therefore, taken together, the elevated SNAI2 and SOX9 protein levels may also have greatly contributed to the up-regulation of cancer stem cell features and motility of those DU145^FP^ cells.

In summary, by a long-term cultivation of prostate cancer DU145 cells in the presence of FP, we have successfully established a FP resistant sub-line that was extensively characterized. Our study provides for the first time sufficient evidence that FP induced mitochondrial lesions stimulate both mitochondrial apoptosis and metabolism reprogramming. The reprogramming and plasticity of cell metabolism can be a fundamental mechanism of induced FP treatment resistance, suggesting that intercepting this process may be a favorable way to overcome the FP resistance in cancer cells.

## Material and Methods

### Cell culture and flavopiridol treatment

Human prostate cancer cell line DU145 (ATCC, VA, USA) was routinely cultured in phenol red free RPMI-1640 medium (Cat. No. 11835–063, Gibco) containing 10% of fetal bovine serum (Gibco), 100 U/ml of penicillin and 100 μg/ml of streptomycin (Gibco). To obtain flavopiridol-tolerant DU145 cells, wild type DU145 cells (DU145^WT^) were maintained in the medium as described above with additional 400 nM flavopiridol hydrochloride (Selleck Chemicals) plus 1 mM sodium pyruvate at final concentration. The flavopiridol-tolerant DU145 cells (DU145^FP^) were routinely cultured in medium containing 400 nM FP and passaged over a half year to stabilize the features. Cells were cultured in a 37 °C saturated incubator containing 5% of CO2 (BINDER). The medium for DU145^FP^ culture, if not specifically mentioned, always contained 400 nM FP in all experiments.

### Apoptosis assays

Cells were collected into 5 ml tubes together with the supernatant. The tubes were centrifuged and the supernatant was carefully removed. The cell pellets were rinsed and then re-suspended in 500 μl binding buffer (Molecular Probes) before being stained by sufficient FITC conjugated Annexin V for 20 minutes in the dark. 5 μl PI (1 mg/ml) were then added and mixed well for another 5 minutes’ incubation. Finally, the cells were washed twice with PBS containing Ca^2+^ and re-suspended in 500 μl DMEM + solution (FluoroBrite DMEM supplemented with 5% of FBS). The cells were analyzed with a LSR II flow cytometer (BD Biosciences). A 488 nm laser was used to excite FITC and a 561 nm laser was applied to excite PI. The data was further analyzed and presented by using software FlowJo version 7.6 (FlowJo).

### Microscopic visualization of mitochondrial membrane potential

The mitochondrial membrane potential (Δψ_m_) sensitive probe JC-1 (Molecular Probes) was prepared into 2 mM stock solution in DMSO. FluoroBrite DMEM (Gibco) medium was used to prepare the medium for staining and live cell imaging. The staining medium contained 10% of FBS, 1 μM JC-1 and 5 μg/ml Hoechst 33342 at final concentration. The imaging medium was only supplemented with 5% of FBS. For live cell imaging, cells were seeded into culture dishes (VWR) until they approximately reached 50% of confluence. For JC-1 staining, the cells were incubated in a pre-warmed staining medium at 37 °C for 20 minutes and kept out of light. Subsequently, the staining medium was discarded, and the cells were washed twice with pre-warmed PBS (Ca^2+^ free). Lastly, sufficient pre-warmed imaging medium was added, and the cells were visualized by a Leica fluorescence imaging system (AF6000 modular system, inverted microscope model DMI6000B, mercury light source EL6000). The JC-1 monomers were presented by a GFP filter set and were displayed in green, and the JC-1 aggregates were visualized by an Y3 filter set and displayed in red. The cell nuclei were co-stained by Hoechst 33342 and visualized by an A4 filter set displayed in blue.

### Metabolic assays

The metabolic status was investigated on an XF^e^96 Extracellular Flux analyzer (Seahorse Bioscience) with standard 96-well Seahorse microplates. A Mito Stress test kit was applied, and the oxygen consumption ratio (OCR) and the extracellular acidification rate (ECAR) were measured. In brief, 2.2*10^4^ cells were seeded into plates 12 hours prior to analysis. The medium was then replaced by 175 µl of non-buffered Dulbecco’s modified Eagle’s medium (DMEM) containing 10 mM glucose, 2 mM glutamine and 1 mM pyruvate. The cells were incubated in a CO_2_-free incubator at 37 °C for one hour to allow for temperature and pH equilibration before being loaded into the XF^e^96 analyzer. The injection sequence was programmed as: 1^st^, oligomycin (1 µM at final concentration); 2^nd^, FCCP (1 µM at final concentration); 3^rd^, rotenone and antimycin A (0.5 µM and 0.5 µM at final concentrations, respectively). The data was analyzed with the software Wave (version 2.2.0, Seahorse Bioscience) for further visual presence.

To determine glucose consumptions, the cells were seeded into 24-well culture plates at 4*10^5^ cells per well and incubated overnight to complete the cell adherence to the plates. The medium was subsequently replaced by 350 µl of fresh normal RPMI-1640 medium (containing 8% FBS) without FP for all cell types. The wells containing only medium without cells were taken as initial controls. For each well at 12, 24 and 48 hours of normal incubation, the supernatants of the cell cultures were transferred into clean 1.5 ml tubes and were frozen under a −20 °C environment. The cell counting was simultaneously performed on an automatic cell counter (Thermo Fisher Scientific) based on the Trypan blue staining assay. Lastly, the glucose concentrations in the medium were measured by using commercial kits (GAHK20, Sigma) and by following the manufacturer’s instructions. A wide band spectrum (340 nm~800 nm) microplate reader (model UVM-340, AYSY) was applied to acquire the OD values at 340 nm for the glucose assays.

For the long-term extracellular acidification measurements, the cells were seeded into 24-well culture plates at 2.2*10^5^ cells per well like the previous description in the above assay. After the cell adherence was complete, the medium was replaced by 500 µl of fresh non-buffered Seahorse DMEM medium, which contained 10 mM of glucose, 2 mM of glutamine and 1 mM of pyruvate. Likewise, the cell culture supernatants were collected at 6, 12, 24 and 48 hours for later pH measurement, and for each time point the cells were counted for quality control. To assess the approximate pH value of the cell culture supernatant samples, we utilized Seahorse sensor cartridges and an inverted fluorescent microscope. First, a series of standard pH solutions was prepared from non-buffered Seahorse DMEM medium. The pH values of these standard solutions were adjusted to pH 4.0, 5.0, 6.0, 7.0 and 8.0 respectively by using a calibrated pH meter (VWR), and 0.5 M NaOH and 0.1% HCl solutions. Next, a Seahorse sensor cartridge was activated (hydrated) overnight following the manufacturer’s instructions. Subsequently, 200 µl for each sample medium and standard solution were loaded into a new Seahorse 96-well cell culture plate. Then, the activated sensor cartridge was removed from its utility plate, quickly and carefully dried on a clean tissue paper tower, and was assembled with the 96-well cell culture plate (contains samples) described in the previous step. Next, the sensor cartridge was maintained with the cell medium samples/standard solutions for about 15 minutes at room temperature in the dark. Lastly, the plate was placed on an inverted fluorescent microscope (AF6000 modular system, inverted microscope model DMI6000B, mercury light source EL6000, Leica). The pH sensors were excited through a GFP filter set and were photographed by camera. For photographing, the exposure time was kept unchanged for every sample/sensor. After the images were captured, the software Image J (version 1.48 v) was used for analyzing fluorescence intensities of the images. The obtained fluorescent intensity data were used for drawing standard curves, and the approximate pH values of the samples were subsequently evaluated. The data presented as mean ± S.D based on two independent experiments, for each experiment two parallel cell samples were included. The intracellular ATP production was tested by an ATP measurement kit (Molecular Probes) based on a bioluminescence assay (firefly luciferase), and the manufacturers’ instructions were strictly followed.

### Assessment of cell proliferation kinetics

DU145 cells were seeded into 6 well plates at a density of 5*10^4^ cells for each well. For each plate, 3 wells were prepared for each cell type. The cell numbers were counted by using Trypan blue assay and an automatic cell counter (Thermo Fisher Scientific) at day 1, 3, 5, 7 and 9.

### Cell cycle analyses

DU145 cells in the logarithmic phase were dissociated by using trypsin, and 2*10^6^ cells were carefully collected. The cells were then fixed in 75% ethanol at 4 °C overnight. The fixed cells were washed twice and re-suspended in 500 μl PBS containing 2 μg/ml RNase A, then incubated at 37 °C for 15 minutes. Next, the cells were centrifuged and re-suspended in 500 μl PBS containing 10 μg/ml PI and incubated for 30 minutes at 4 °C in the dark. The cells were then washed and prepared into single cell suspensions before undergoing further analysis on an LSR II flow cytometer (BD Biosciences).

### Flow cytometry analyses of integrin, EPCAM, CD44 and ABCG2

To examine the expression of integrin α2β3, the cells were fixed in 4% PFA-PBS solution at 4 °C overnight. The fixed cells were washed with cold PBS and re-suspended in sufficient blocker solution (0.5% of IgG-free BSA, 0.1% in PBS) for 30 minutes at room temperature, then the cell pellets were collected. The cells were re-suspended in the blocker solution contains primary antibody that against integrin α2β3 (dilution of 1:1000) and incubated for 2 hours at room temperature, then the cells were washed and incubated with Alexa Fluor 488 conjugated secondary antibody (dilution of 1:2000) for another 1 hour in the dark. At last the cells were washed with cold PBS and re-suspended with blocker solution.

To examine the CD44, ABCG2 and EPCAM, the cells were carefully disassociated by using TrypLE Express (Gibco) and prepared into single cell suspensions. For each test, 1*10^6^ of cells were prepared in 5 ml tubes and washed by cold PBS. The cells were centrifuged and re-suspended in sufficient blocker solution, which was prepared with 0.5% of IgG-free BSA in FluoroBrite DMEM medium (Gibco), at 37 °C for 15 minutes before the supernatant was discarded. The cells were then stained with antibodies or isotype control antibodies according to the instructions supplied by the manufacturers. Before passing through the flow cytometer, the cells were filtered through 70-μm cell strainers, and sufficient PI solution was added to exclude dead cells (not applied for fixed cells). Samples were analyzed on a BD LSRII flow cytometer, and Software FlowJo version 7.6 was used for further data analysis. All above experiments were repeated three times.

The applied antibodies are as follows: anti-CD44 (559942, BD Bioscience), anti-ABCG2 (561451, BD Bioscience), anti-EPCAM (347200, BD Bioscience), anti-integrin α2β3 (ab662, Abcam), Alexa Fluor 488 conjugated goat anti-Mouse IgG secondary antibody (A-11001, Thermo Fisher Scientific).

### Side population assays

All of the cells were routinely cultured up to 60% confluence in 100 mm diameter dishes and were disassociated by using TrypLE reagent (Gibco). Approximately 1*10^6^ of cells were collected and maintained in pre-warmed DMEM+ solution containing 2% of fetal bovine serum prepared by DMEM medium (HEPES added)(Gibco). Hoechst 33342 stock solution (1 mg/ml) was then added to a final concentration of 5 μg/ml and mixed well. The cell suspensions were incubated with intermittent shaking for 90 minutes in a 37 °C water bath and kept from light. As staining controls, cells were stained as described above but with an extra 50 μM verapamil at final concentration. After incubation, the cells were washed with ice-cold HBSS+ solution (HBSS buffer (Gibco) containing 2% of FBS and centrifuged at 4 °C. The cells were re-suspended in ice-cold HBSS+ and placed on ice until examined on the flow cytometer. Before passing through the flow cytometer, the cells were filtered through a 70-μm cell strainer and sufficient PI solution was added to detect dead cells. The Hoechst 33342 dye was excited with a UV laser at 350 nm. The Hoechst blue signal was detected with a 450/50 filter set, and the Hoechst red signal was detected with a 610/20 filter set. The protocol published by Goodell *et al*. was used as the main reference for this assay^95^.

### Rhodamine 123 efflux assay

The procedure of rhodamine 123 efflux assay in this study was generally referred to the method published by Laupèze B *et* *al*.^96^ with a minor modification. DU145 cells were cultured in 6-well dish until 60–80% confluence. To load the cells with rhodamine 123, cells were maintained in phenol red-free RPMI-1640 medium, which was supplemented with 5% FBS and 0.5 µg/mL rhodamine 123 at final concentration. The control cells were maintained in staining medium with additional 100 µM verapamil at final concentration. Then the cells were stained for 30 minutes in the dark. After that, staining medium was removed, and the cells were carefully washed with pre-cold DPBS (Ca^2+^ free), then the cells were dissociated by using 0.25% trypsin-EDTA solution. Cell suspensions were centrifuged to collect cell pellets, and the cells were then re-suspended in 1 ml pre-warmed normal RPMI-1640 media with or without 100 µM verapamil, in accordance with the staining samples or control samples respectively. The cell suspensions were incubated in a water bath adjusted to 37 °C, and the fluorescence intensity of rhodamine 123 within each cell sample was assessed by using a BD LSR II flow cytometer after every one hour incubation (after each time flow cytometry detection, the remaining cells were placed back to the water bath until the experiment was finished). The rhodamine 123 was excited by a 488 nm laser and the fluorescence signals were detected from the PMT channel with 530/30 band pass filter. Further data analyses were accomplished by using software FlowJo (version 10.0.7).

### Chemotherapy

Cells were treated with docetaxel and cisplatin for 24–96 hours, and the cell viabilities were determined every 24 hours. For the concentrations, 0 nM (DMSO control), 10 nM and 20 nM docetaxel and 0 μM (DMSO control), 30 μM and 60 μM cisplatin were applied. The cell viability assays were based on Annexin V-FITC (Molecular Probes) and PI (Molecular Probes) co-staining. The cytometer configurations were exactly the same as described above for other flow cytometry assays, and the software FlowJo (version 7.6) was used for further data analysis.

### Transwell assay

Six thousand (DU145) cells were prepared in serum-free medium and loaded into 8 μm pore polycarbonate membrane inserts (Transwell, Corning). The plate wells were filled with 500 μl medium containing 10% of FBS and 200 ng/ml G-CSF. After 24 hours of incubation, the insert chambers were fixed with methanol and stained by 0.1% of crystal violet (Sigma) in methanol (VWR). The unpenetrated cells were carefully removed with cotton swabs and the polycarbonate membranes were dried under room temperature. Lastly, the membranes were cut and mounted in slides, and the absolute cell number after penetration was manually counted in a blinded manner. To estimate the number of total penetrated cells, slides were photographed under a microscope connected with digital camera. For each membrane, 10 random microscopic field were recorded, and the obtained cell counts were applied to estimate the total penetrated cells on the membrane.

### Cell wound healing assays

The DU145 cells were seeded into 60-mm dishes and maintained until nearly 100% confluence. Then, sterilized 10 μl pipette tips were used to make scratches on the single cell layers. Immediately after the dishes were placed into a real-time imaging system manufactured by our laboratory. Images were photographed every three minutes by a Canon DSLR model 600D until the scratches healed. The healing processes were followed via time-lapse videos based on the captured images. Each second in playback equaled to 25 minutes of real time. The average scratch width was measured by using software Adobe Photoshop CS6 extended version (version 13.0.1), and the obtained data was used for further calculation of the relative healing speed.

### F-actin staining

The F-actin was visualized by staining the DU145 cells with rhodamine conjugated phalloidin (Molecular Probes). The cells were cultured on cover slides in six well plates and fixed with a 4% formaldehyde solution in PBS for 10 minutes at room temperature. The slides were washed twice with PBS, and then treated with 0.1% Triton X-100 in PBS solution for 5 minutes. Next, the slides were washed twice with PBS, and incubated in 1% BSA-PBS solution for 20 minutes to block unspecific binding sites. The cells were subsequently stained with 2 U/ml rhodamine conjugated phalloidin and 5 μg/ml DAPI prepared in PBS solution for 20 minutes in the dark. Finally, the cover slides were carefully washed and mounted. The cells’ visualization and photography were performed on a Nikon ECLIPSE E600 fluorescent microscope with a customized optical adaptor with camera. The microscopy fields were randomly selected.

### Transcriptome analyses

Cells were cultured in 100-mm dishes until approximately 60% confluent. Next, the culture medium was removed, and the cells were washed twice with cold PBS before the total intracellular RNA was isolated by using a commercial mRNA isolation kit (Invitrogen). The isolation instructions supplied by the manufacturer were strictly followed. All of the working space was pre-cleaned by RNase Away regent (Thermo Fisher Scientific), and an additional on-column DNA digestion procedure was applied by using a PureLink DNase kit (Thermo Fisher Scientific). The quality of the RNA samples was evaluated on a Nanodrop 2000 UV-Vis Spectrophotometer (Thermo Fisher Scientific). Further RNA quality controls were carried out by BGI Hong Kong before undergoing sequencing. RNA-Sequencing experiments were performed by BGI followed a standard protocol and were sequenced using Illumina HiSeqTM 2000. Raw reads filtering, quality control, alignment of cleaned reads to human reference genome HG19, and genomic annotations were carried out at BGI based on a previous publication^97^. In total genomic reads, there are ~45 millions unique matches (~80%) in each sample. Gene expression level was calculated by the Fragments Per Kilobase Per Million Fragments Mapped (FPKM)^97^. Subsequently, FPKM values were used in further data analysis, where an algorithm based on “The significance of digital gene expression profiles”^98^ was used to identify differentially expressed genes between two samples. P-value corresponding to differential gene expression test was corrected by False Discovery Rate (FDR) method^99^. The P-value < 0.05 and FDR < 0.001 was applied as the threshold to judge the significance of gene expression difference.

The genes listed in the heat maps were manually selected from the dataset of the transcriptome analysis, based on the references previously published by other researchers, company or on line resources. The functions of apoptosis related genes were referred to the information provided by the Cell Signaling Company (https://www.cellsignal.com/common/content/content.jsp?id=pathways-apoptosis-control). The gene functions were confirmed in the references attached therewith, and were further confirmed in the on line database GeneCards (http://www.genecards.org/). The genes included in the cell cycle regulations were referred to the previously published references^100–102^. The ETC related genes were selected based on the results of “GO function” provided in the dataset per se.

### Immunoblotting

Cells were lysed in freshly prepared RIPA buffer supplemented with 1x Halt Protease/Phosphatase Inhibitor Cocktail (Thermo Fisher Scientific). Cleared lysates were resolved by SDS-PAGE, transferred to PVDF membrane and incubated with primary antibodies. The antibodies applied in the current study are as follows: anti-BCL-2 (M0887, DAKO), anti-BID (#2002, Cell Signaling Technology), anti-BAG3 (NBP2-27398, NOVUS), anti-BOK (AF6067, R&D System), anti-BAK (SC-7873, Santa Cruz), anti-BAD (610391, BD Bioscience), anti-CASP9 (#9502, Cell Signaling Technology), anti-CYCS (556433, BD Bioscience), anti-BIRC5 (SURVIVIN) (#2802, Cell Signaling Technology), anti-XIAP (#2042, Cell Signaling Technology), GAPDH (AF5718, R&D System), anti-SNAI2 (SLUG) (ab27568, Abcam), anti-SOX9 (ab26414, Abcam), anti-total CD44 (ab51037, Abcam), anti-integrin α2β3 (ab662, Abcam), anti-HIF1A (AF1935, R&D System), anti-HIF2A (AF2886, R&D System) and α-tubulin (T5168, Sigma).

### Statistical analyses

For the experiments listed below at least three independent experiments were performed, and the data were presented as mean ± S.D. The related statistical significances were obtained from performing 2-tail Student’s t-test. The mentioned experiments are including: mitochondrial membrane potential evaluation (Fig. 2A); apoptosis assay (Figs 2B and S4); ATP assay (Fig. 3A); glucose consumption test (Fig. 3B); measurement of pH values (Fig. 3B); cell cycle analysis (Fig. 3D); trans-well assay (Fig. 4A); wound healing assay (Fig. 4A); the flow cytometry detection of ABCG2, side population, rhodamine 123 efflux (Fig. 6); MitoSox Red staining (Fig. S3B).

For multiple-group comparison of the data obtained from three independent experiments in cisplatin and docetaxel chemotherapy treatments (Fig. 5), one-way ANOVA test was applied (Bonferroni method). Statistical significance is represented in figures by *p < 0.05, **p < 0.01, ***p < 0.001. All analyses were performed with the SPSS version 20 statistics software (IBM).

## Electronic supplementary material

SV1

SV2

SV3

Supplementary Material
