# Supplementary material for: Metabolic reprogramming is associated with flavopiridol resistance in prostate cancer DU145 cells
Source: Sci Rep. 2017 Jul 11;7:5081. doi: 10.1038/s41598-017-05086-6 (PMC5506068; doi:10.1038/s41598-017-05086-6)
Supplement: Supplementary file 4 — Supplementary Material [file 41598_2017_5086_MOESM4_ESM.pdf]

Supplemental Material for Manuscript:

Metabolic reprogramming is associated with flavopiridol resistance in prostate cancer DU145 cells

Xiaoran Li[1, 2], Jie Lu[3], Quancheng Kan[4], Xiaoli Li[5], Qiong Fan[6], Yaqing Li[5], Ruixia Huang[7], Ana Slipicevic[1], Hiep Phuc Dong[1], Lars Eide[8], Junbai Wang[1], Hongquan Zhang[9], Viktor Berge[10], Mariusz Adam Goscinski[11], Gunnar Kvalheim[12], Jahn M.Nesland[1, 2], Zhenhe Suo[1, 2*]

[1]Department of Pathology, The Norwegian Radium Hospital, Oslo University Hospital, Oslo, 0379, Norway

[2]Department of Pathology, Institute of Clinical Medicine, Faculty of Medicine, University of Oslo, Oslo, 0318, Norway

[3]Department of Epidemiology and Biostatistics, College of Public Health, Zhengzhou University, Zhengzhou, Henan, 450001, China

[4]Department of Clinical Pharmacology, The First Affiliated Hospital of Zhengzhou University, Zhengzhou, Henan 450001, China

[5]Department of Oncology, The First Affiliated Hospital of Zhengzhou University, Zhengzhou University, Zhengzhou, Henan, 450052, China

[6]Department of Nutrition, Institute of Basic Medical Sciences, University of Oslo, Oslo, 0316, Norway

[7]Department of Radiation Biology, Institute for Cancer Research, Oslo University Hospital, 0379, Norway

[8]Department of Medical Biochemistry, University of Oslo and Oslo University Hospital, Oslo, 0372, Norway

[9]Laboratory of Molecular Cell Biology and Tumor Biology, Department of Anatomy, Histology and Embryology, Peking University Health Science Center, Beijing, 100191, China

[10]Department of Urology, The Norwegian Radium Hospital, Oslo University Hospital, Oslo, 0379, Norway.

[11]Departments of Surgery, The Norwegian Radium Hospital, Oslo University Hospital, Institute for Clinical Medicine, Faculty of Medicine, University of Oslo, Oslo, 0379, Norway

[12]Department of Cell Therapy, Cancer Institute, The Norwegian Radium Hospital, Oslo University Hospital, Oslo, 0379, Norway

*Corresponding author: Dr. Zhenhe Suo, Dept. of Pathology, The Norwegian Radium Hospital, Oslo University Hospital, University of Oslo, Montebello, Ullernchausseen 70, N-0379, Oslo, Norway. E-mail: zhenhes@medisin.uio.no

Figure S1

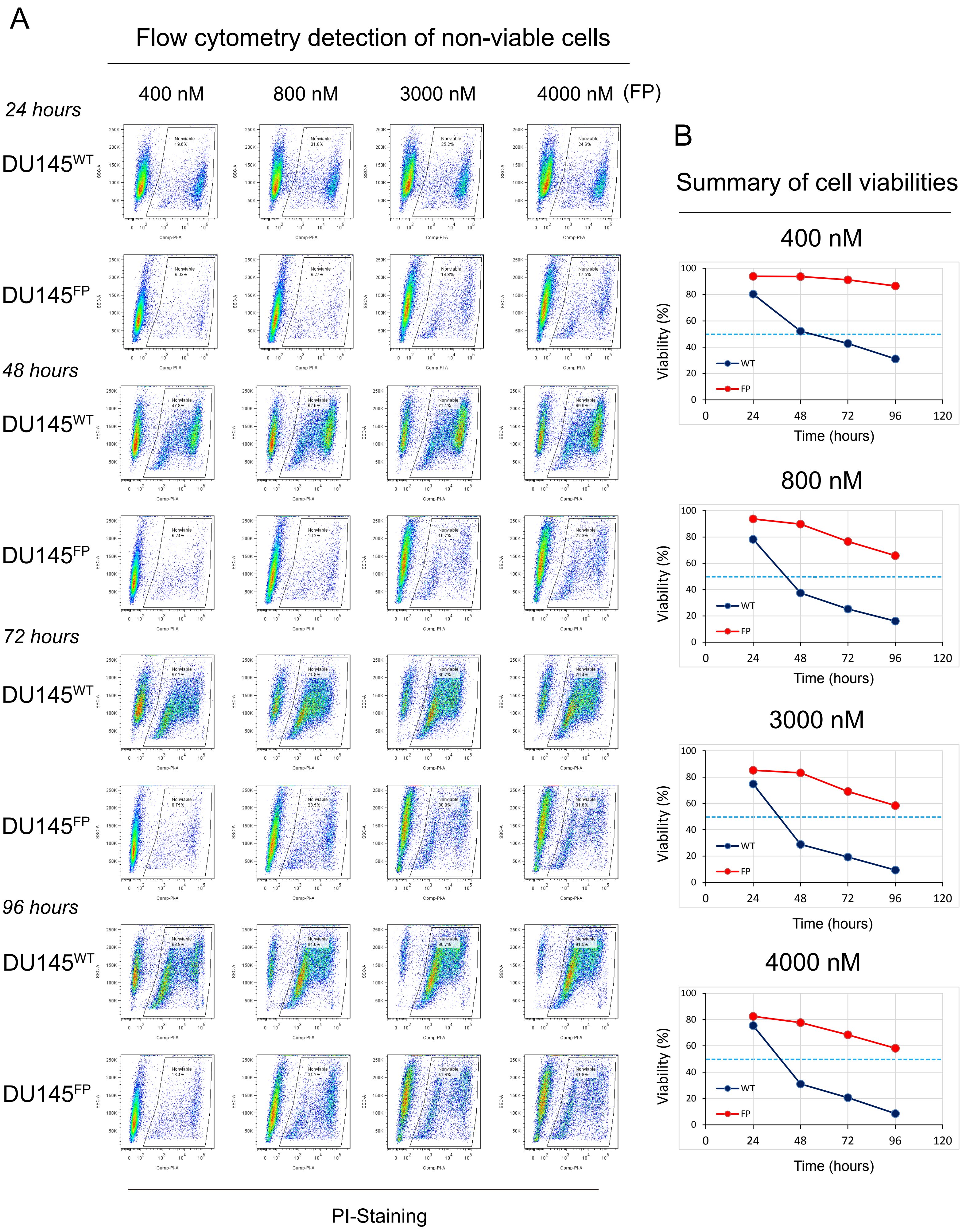

A
Flow cytometry detection of non-viable cells
400 nM
800 nM
3000 nM
4000 nM (FP)
24 hours
48 hours
72 hours
96 hours
DU145WT
DU145FP
PI-Staining
B
Summary of cell viabilities
400 nM
800 nM
3000 nM
4000 nM
Viability (%)
Time (hours)
WT
FP

Figure S2

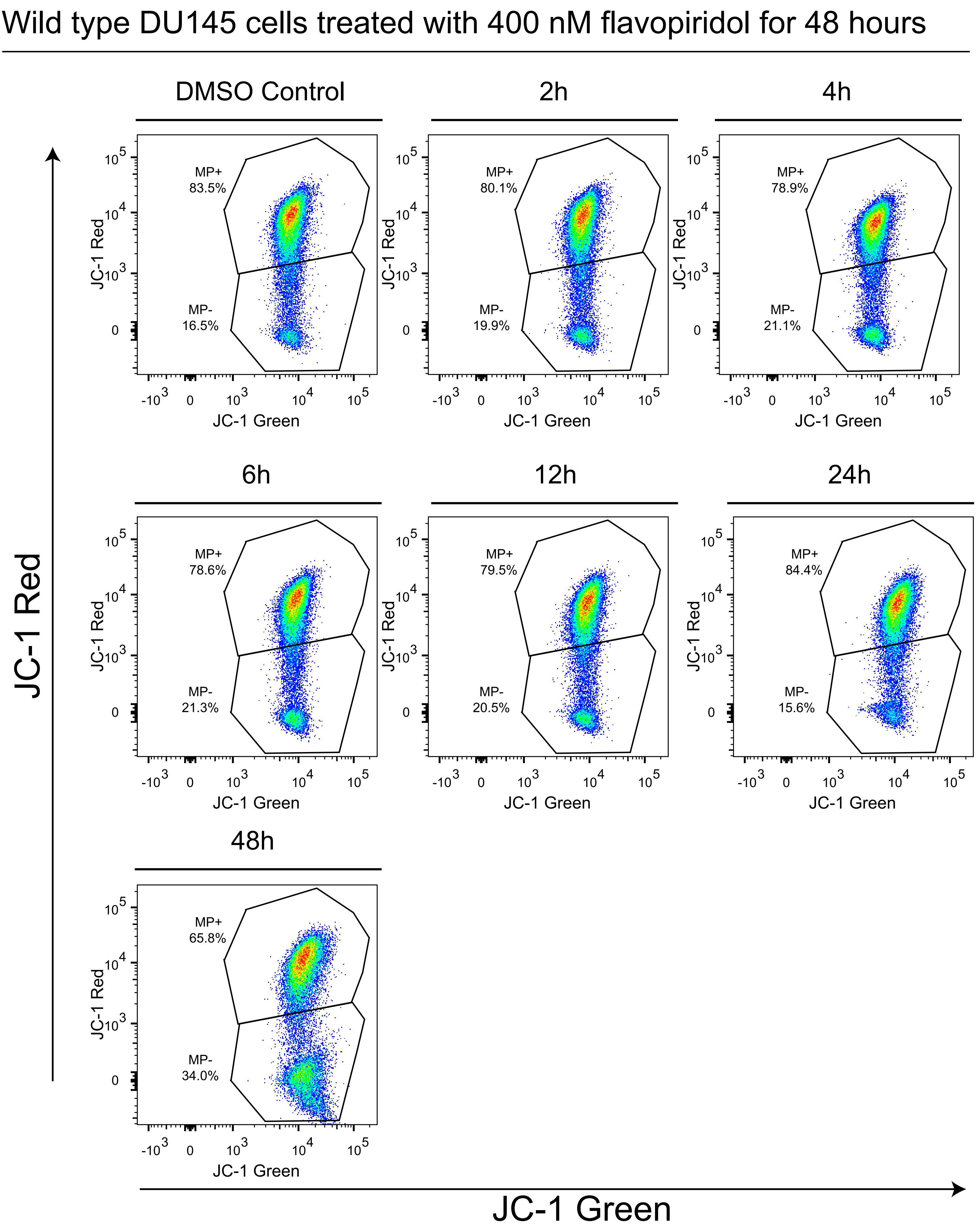
Wild type DU145 cells treated with 400 nM flavopiridol for 48 hours
DMSO Control
MP+ 83.5%
MP- 16.5%
2h
MP+ 80.1%
MP- 19.9%
4h
MP+ 78.9%
MP- 21.1%
6h
MP+ 78.6%
MP- 21.3%
12h
MP+ 79.5%
MP- 20.5%
24h
MP+ 84.4%
MP- 15.6%
48h
MP+ 65.8%
MP- 34.0%
JC-1 Red
JC-1 Green

Figure S3

A

Complex I

Gene Symbol	FP vs. WT	P	FDR
NDUFA4L2		NS	0.64
NDUFAF3		***	0.00
NDUFV3		***	0.00
NDUFB3		***	0.00
NDUFA2		***	0.00
NDUFB5		***	0.00
NDUFA10		***	0.00
NDUFAF2		*	0.06
ND3		***	0.00
NDUFS8		***	0.00
NDUFA7		*	0.10
NDUFB7		NS	0.35
NDUFA6		NS	0.16
NDUFC2		NS	0.13
NDUFV1		**	0.01
NDUFS5		NS	0.14
NDUFS1		*	0.05
ND2		***	0.00
NDUFS3		NS	0.35
NDUFA5		NS	0.65
NDUFB4		NS	0.59
NDUFA1		NS	0.65
NDUFS7		NS	0.64
ND4		NS	0.73
NDUFB11		NS	0.93
NDUFA13		NS	0.86
NDUFB8		NS	0.74
NDUFAF5		NS	0.84
ND4L		***	0.02
NDUFB2		NS	0.65
NDUFB1		NS	0.64
NDUFAB1		NS	0.57
NDUFS4		NS	0.69
NDUFS6		NS	0.37
NDUFAF1		NS	0.63
NDUFA9		NS	0.38
NDUFA11		*	0.09
NDUFC1		NS	0.39
NDUFA3		NS	0.40
NDUFV2		**	0.01
NDUFS2		***	0.00
NDUFAF7		NS	0.26
NDUFA4		***	0.00
NDUFA12		*	0.05
NDUFAF6		NS	0.17
ND5		***	0.00
NDUFB6		*	0.00
NDUFA8		***	0.00
ND6		***	0.00
ND1		***	0.00
NDUFAF4		***	0.00
NDUFB9		***	0.00
NDUFB10		***	0.00
NDUFAF4P1		NS	0.61

Complex II

Gene Symbol	FP vs. WT	P	FDR
SDHD		*	0.00
SDHC		NS	0.21
SDHB		*	0.05
SDHA		***	0.00

Complex III

Gene Symbol	FP vs. WT	P	FDR
UQCRC1		***	0.00
UQCRQ		*	0.05
CYTB		***	0.00
UQCR10		NS	0.20
UQCRFS1		NS	0.12
UQCRC2		NS	0.91
UQCR11		NS	0.63
UQCRH		*	0.03
CYC1		***	0.00
UQCRB		***	0.00

Complex IV

Gene Symbol	FP vs. WT	P	FDR
COX3		***	0.00
COX6B1		**	0.01
COX4I1		**	0.01
COX7B		NS	0.27
COX7A2		NS	0.44
COX5B		NS	0.58
COX2		***	0.00
COX8A		*	0.06
COX6A1		*	0.05
COX6B2		NS	0.79
COX1		***	0.00
COX5A		***	0.00
COX6C		***	0.00
COX7C		***	0.00

Complex V

Gene Symbol	FP vs. WT	P	FDR
ATP5B		***	0.00
ATP5D		***	0.00
ATPAF1		*	0.08
ATP5A1		***	0.00
ATP5G1		*	0.09
ATP6		***	0.00
ATP5F1		NS	0.33
ATP5G3		NS	0.35
ATP5E		NS	0.85
ATP5C1		NS	0.67
ATP5G2		***	0.00
ATPAF2		*	0.04

MtDNA encoded genes

Gene Symbol	FP vs. WT	P	FDR
ND3		***	0.00
COX3		***	0.00
CYTB		***	0.00
ATP6		***	0.00
ND2		***	0.00
ATP8		**	0.01
ND4		NS	0.73
ND4L		**	0.02
COX2		***	0.00
COX1		***	0.00
ND5		***	0.00
ND6		***	0.00
ND1		***	0.00

Color Index Log2(Folds)

-1	-0.25	0	0.25	1

B

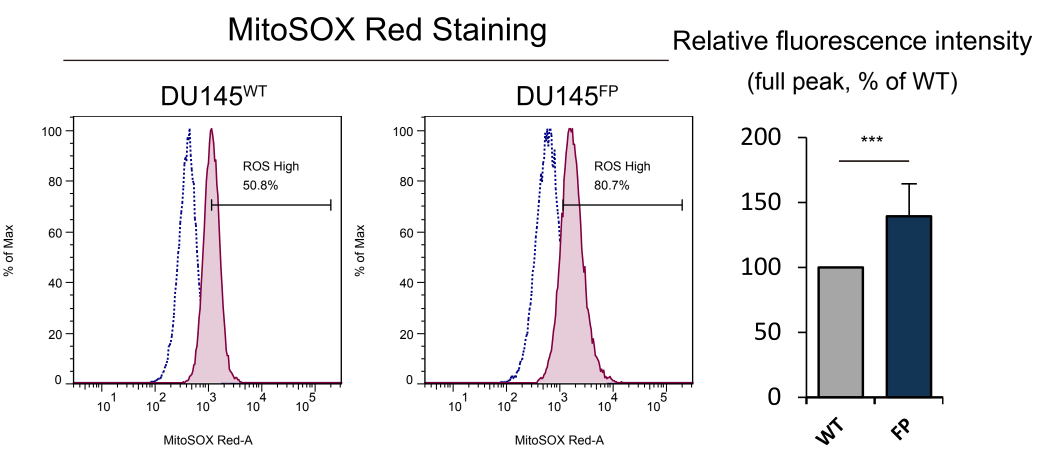

Figure S4

A

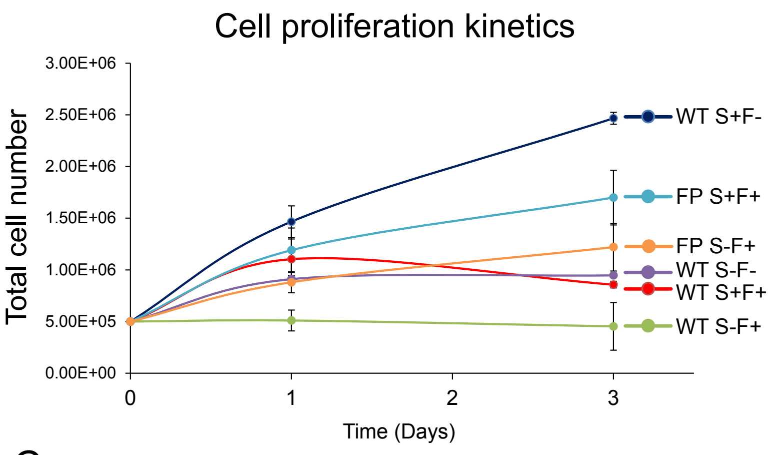

B

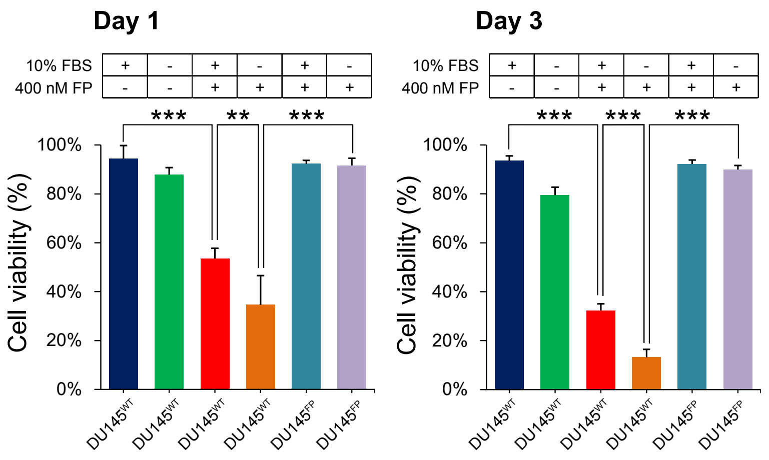

C

Day 1

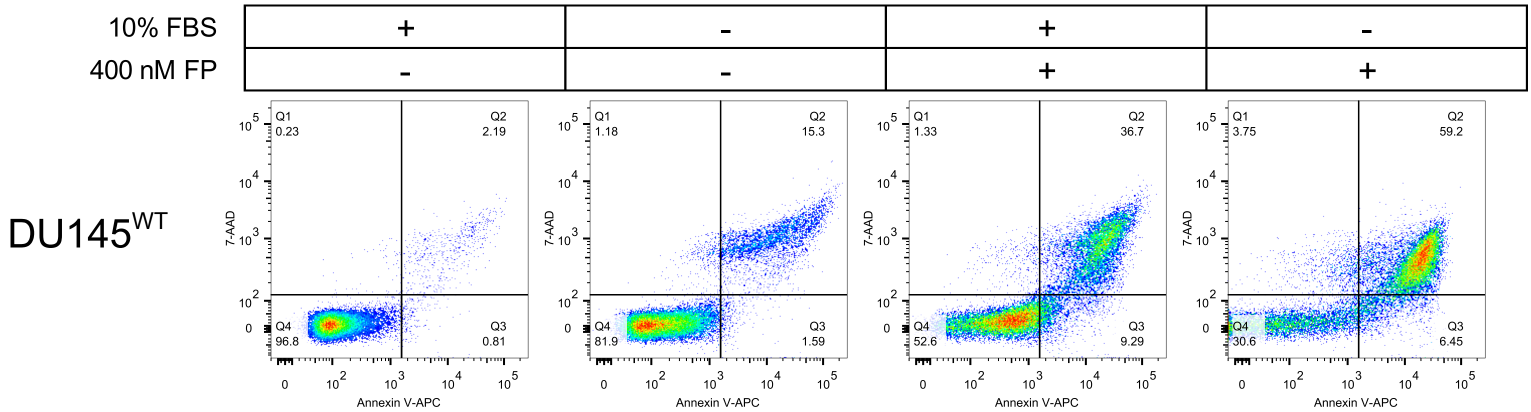

Cell viability

10% FBS	+	-	+	-
400 nM FP	-	-	+	+
DU145WT	~94.4%	~87.9%	~53.5%	~34.6%
DU145FP	/	/	~92.3%	~91.6%

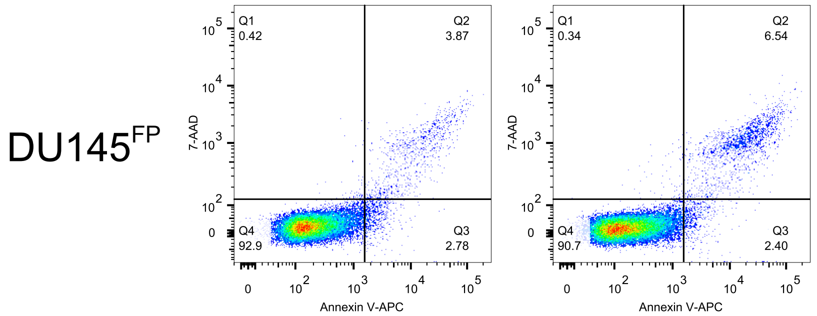

Day 3

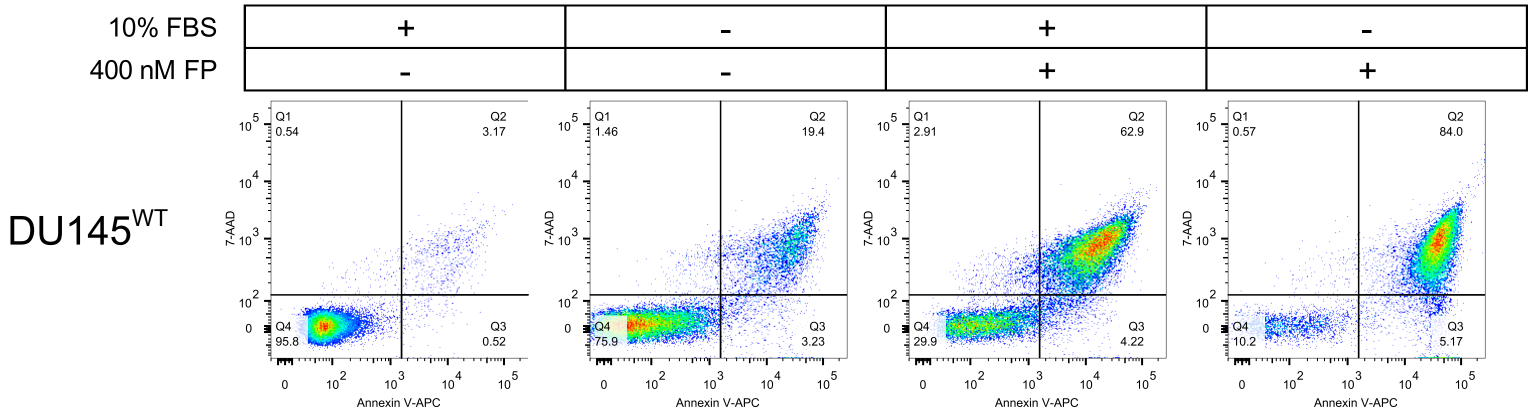

Cell viability

10% FBS	+	-	+	-
400 nM FP	-	-	+	+
DU145WT	~93.5%	~79.5%	~32.3%	~13.2%
DU145FP	/	/	~92.2%	~89.9%

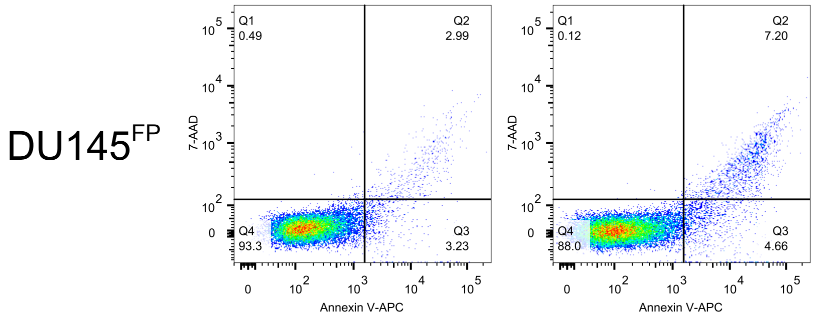

Figure S5

Index:A

Aliases	Gene Symbol	Folds	FP vs. WT	P	FDR
AIF	*AIFM1*	0.85		**	0.01
	AIFM2	0.98		NS	0.80
	AIFM3	0.83		NS	0.31

Aliases	Gene Symbol	Folds	FP vs. WT	P	FDR
AKT	*AKT1*	1.49		***	0.00
	AKT2	0.84		***	0.00
	AKT3	1.46		***	0.00

Aliases	Gene Symbol	Folds	FP vs. WT	P	FDR
APAF1	*APAF1*	1.94		***	0.00

Aliases	Gene Symbol	Folds	FP vs. WT	P	FDR
ARTS	*PRPS1*	0.64		***	0.00
	PRPS2	1.36		***	0.00

Aliases	Gene Symbol	Folds	FP vs. WT	P	FDR
ATM	*ATM*	0.86		**	0.01

Aliases	Gene Symbol	Folds	FP vs. WT	P	FDR
ATR	*ATR*	1.08		NS	0.19

Index:B

Aliases	Gene Symbol	Folds	FP vs. WT	P	FDR
BAD	*BAD*	1.18		**	0.00

Aliases	Gene Symbol	Folds	FP vs. WT	P	FDR
BAG	*BAG1*	1.27		***	0.00
	BAG2	0.91		NS	0.11
	BAG3	1.28		***	0.00
	BAG4	0.78		**	0.01
	BAG5	0.98		NS	0.87
	BAG6	0.85		***	0.00

Aliases	Gene Symbol	Folds	FP vs. WT	P	FDR
BAK	*BAK1*	0.78		***	0.00

Aliases	Gene Symbol	Folds	FP vs. WT	P	FDR
BAX	*BAX*	0.93		NS	0.30

Aliases	Gene Symbol	Folds	FP vs. WT	P	FDR
BCL-XL	*BCL2L1*	1.14		***	0.00

Aliases	Gene Symbol	Folds	FP vs. WT	P	FDR
BCL2	*BCL2*	2.32		***	0.00

Aliases	Gene Symbol	Folds	FP vs. WT	P	FDR
BID	*BID*	1.73		***	0.00

Aliases	Gene Symbol	Folds	FP vs. WT	P	FDR
BIM	*BCL2L11*	1.19		NS	0.95

Index:C

Aliases	Gene Symbol	Folds	FP vs. WT	P	FDR
Calcineurin	*PPP3CC*	0.48		***	0.00

Aliases	Gene Symbol	Folds	FP vs. WT	P	FDR
CAMKII	*CAMK2A*	0.03		NS	0.39
	CAMK2B	1.53		**	0.02
	CAMK2D	1.04		NS	0.61
	CAMK2G	1.25		***	0.00

Aliases	Gene Symbol	Folds	FP vs. WT	P	FDR
CASPASE 2	*CASP2*	1.09		NS	0.23

Aliases	Gene Symbol	Folds	FP vs. WT	P	FDR
CASPASE 3	*CASP3*	1.21		NS	0.11

Aliases	Gene Symbol	Folds	FP vs. WT	P	FDR
CASPASE 9	*CASP9*	0.48		***	0.00

Aliases	Gene Symbol	Folds	FP vs. WT	P	FDR
CASPASE 8	*CASP8*	0.90		NS	0.63

Aliases	Gene Symbol	Folds	FP vs. WT	P	FDR
CASPASE 10	CASP10	0.93		NS	0.81

Aliases	Gene Symbol	Folds	FP vs. WT	P	FDR
Cyto C	*CYC1*	0.87		***	0.00
	CYCS	0.88		***	0.00

Index:D-F

Aliases	Gene Symbol	Folds	FP vs. WT	P	FDR
DIABLO	*DIABLO*	0.96		NS	0.94

Aliases	Gene Symbol	Folds	FP vs. WT	P	FDR
ENDOG	*ENDOG*	0.96		NS	0.79

Aliases	Gene Symbol	Folds	FP vs. WT	P	FDR
ERK1	*MAPK3*	0.97		NS	0.68

Aliases	Gene Symbol	Folds	FP vs. WT	P	FDR
ERK2	*MAPK1*	1.30		***	0.00

Aliases	Gene Symbol	Folds	FP vs. WT	P	FDR
FAS	*FAS*	1.14		NS	0.52

Aliases	Gene Symbol	Folds	FP vs. WT	P	FDR
FADD	*FADD*	1.19		**	0.01

Index:H

Aliases	Gene Symbol	Folds	FP vs. WT	P	FDR
HRK	*HRK*	4.64		***	0.00

Aliases	Gene Symbol	Folds	FP vs. WT	P	FDR
HSP60	*HSPD1*	0.90		***	0.00

Aliases	Gene Symbol	Folds	FP vs. WT	P	FDR
HTRA2	*HTRA2*	1.39		***	0.00

Index:I-N

Aliases	Gene Symbol	Folds	FP vs. WT	P	FDR
ING2	*ING2*	0.71		NS	0.13

Aliases	Gene Symbol	Folds	FP vs. WT	P	FDR
JNK	*MAPK8*	0.67		***	0.00

Aliases	Gene Symbol	Folds	FP vs. WT	P	FDR
LC8	*DYNLL1*	1.04		NS	0.40
	DYNLL2	1.10		NS	0.23

Aliases	Gene Symbol	Folds	FP vs. WT	P	FDR
MULE	*HUWE1*	1.09		***	0.00

Aliases	Gene Symbol	Folds	FP vs. WT	P	FDR
MCL1	*MCL1*	0.74		***	0.00

Aliases	Gene Symbol	Folds	FP vs. WT	P	FDR
NOXA	*PMAIP1*	2.99		***	0.00

Index:P

Aliases	Gene Symbol	Folds	FP vs. WT	P	FDR
P53	*TP53*	1.30		***	0.00

Aliases	Gene Symbol	Folds	FP vs. WT	P	FDR
p70 S6K	*RPS6KB1*	1.14		*	0.03
	RPS6KB2	1.34		***	0.00

Aliases	Gene Symbol	Folds	FP vs. WT	P	FDR
p90 RSK	*RPS6KA1*	0.83		***	0.00
	RPS6KA2	2.40		***	0.00
	RPS6KA3	0.93		NS	0.20
	RPS6KA4	1.21		***	0.00
	RPS6KA5	1.08		NS	0.63
	RPS6KA6	0.91		NS	0.62

Aliases	Gene Symbol	Folds	FP vs. WT	P	FDR
PI3K	*PIK3R1*	0.81		NS	0.11
	PIK3R2	1.16		**	0.03
	PIK3R3	0.74		**	0.01
	PIK3R4	0.90		NS	0.16
	PIK3R5	0.11		***	0.00

Aliases	Gene Symbol	Folds	FP vs. WT	P	FDR
PIDD	*PIDD1*	1.23		*	0.09

Aliases	Gene Symbol	Folds	FP vs. WT	P	FDR
PKA	*PRKACA*	1.11		**	0.01
	PRKACB	2.90		***	0.00

Aliases	Gene Symbol	Folds	FP vs. WT	P	FDR
PKC	*PRKCA*	1.01		NS	0.83
	PRKCB	0.03		NS	0.64
	PRKCD	1.10		NS	0.34
	PRKCDBP	3.89		***	0.00
	PRKCE	0.74		*	0.04
	PRKCG	0.64		NS	0.28
	PRKCH	1.21		NS	0.39
	PRKCI	0.88		***	0.00
	PRKCQ	0.64		**	0.00
	PRKCZ	1.16		*	0.07

Index:P-X

Aliases	Gene Symbol	Folds	FP vs. WT	P	FDR
PUMA	*BBC3*	1.35		NS	0.53

Aliases	Gene Symbol	Folds	FP vs. WT	P	FDR
RAIDD	*CRADD*	1.00		NS	1.00

Aliases	Gene Symbol	Folds	FP vs. WT	P	FDR
SIRT2	*SIRT2*	1.36		***	0.00

Aliases	Gene Symbol	Folds	FP vs. WT	P	FDR
XIAP	*XIAP*	0.96		NS	0.33

Index:Other

Aliases	Gene Symbol	Folds	FP vs. WT	P	FDR
14-3-3	*YWHAQ*	1.05		*	0.05

Color Index Log2(Ratio)						
-2	-1	-0.5	0	0.5	1	2

Figure S6

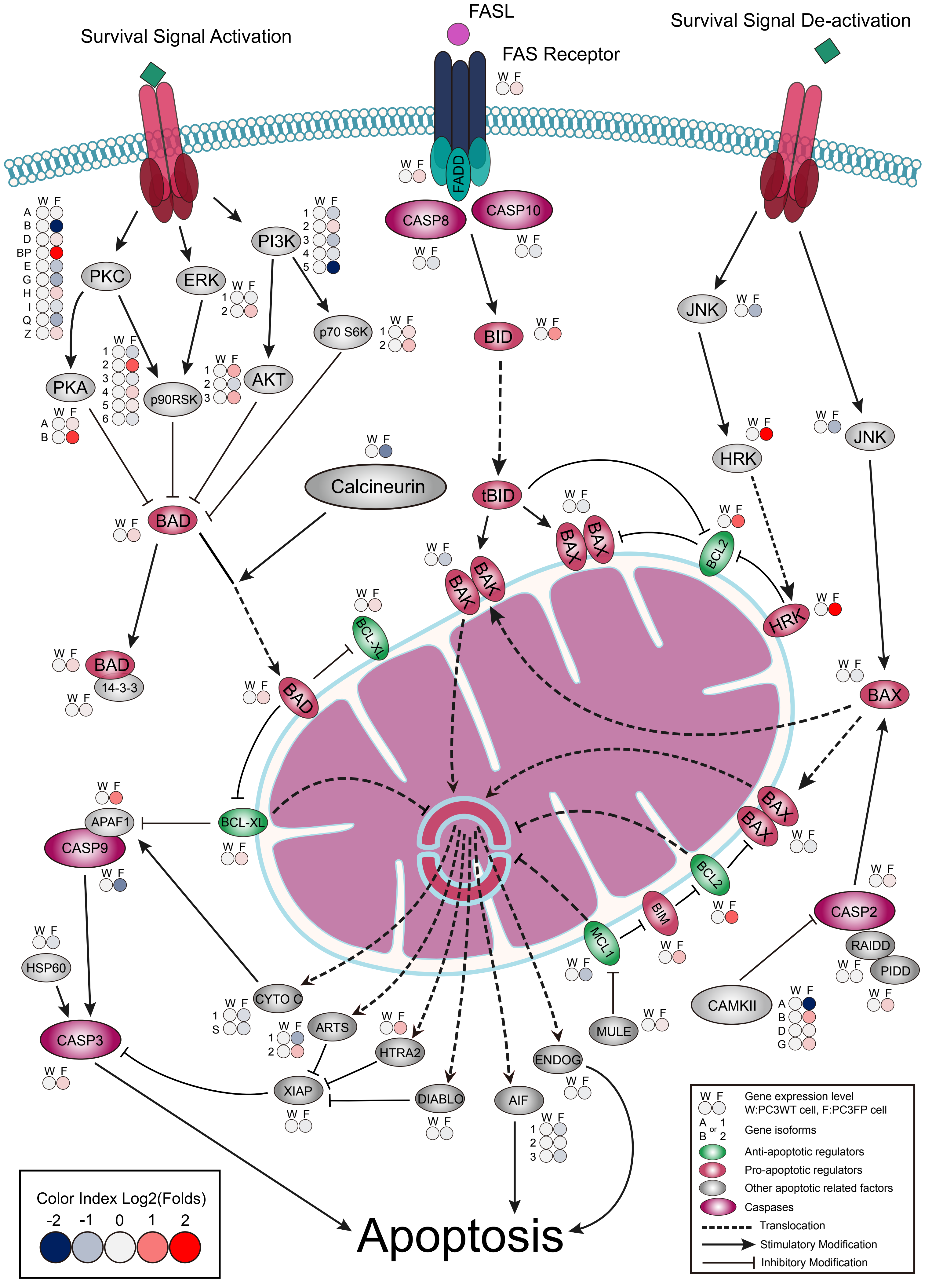

Survival Signal Activation
FASL
FAS Receptor
FADD
Survival Signal De-activation
PKC
ERK
PI3K
CASP8
CASP10
p70 S6K
BID
JNK
PKA
p90RSK
AKT
HRK
JNK
Calcineurin
tBID
BAD
BAX
BAX
BCL2
BAK
BAK
BCL-XL
HRK
BAD
14-3-3
BAD
BAX
APAF1
CASP9
BCL-XL
BAX
BAX
BCL2
BIM
MCL1
CASP2
RAIDD
PIDD
HSP60
CYTO C
ARTS
HTRA2
MULE
CAMKII
CASP3
XIAP
DIABLO
AIF
ENDOG
Apoptosis
Color Index Log2(Folds)
-2 -1 0 1 2
Gene expression level
W:PC3WT cell, F:PC3FP cell
Gene isoforms
Anti-apoptotic regulators
Pro-apoptotic regulators
Other apoptotic related factors
Caspases
Translocation
Stimulatory Modification
Inhibitory Modification

Figure S7

A

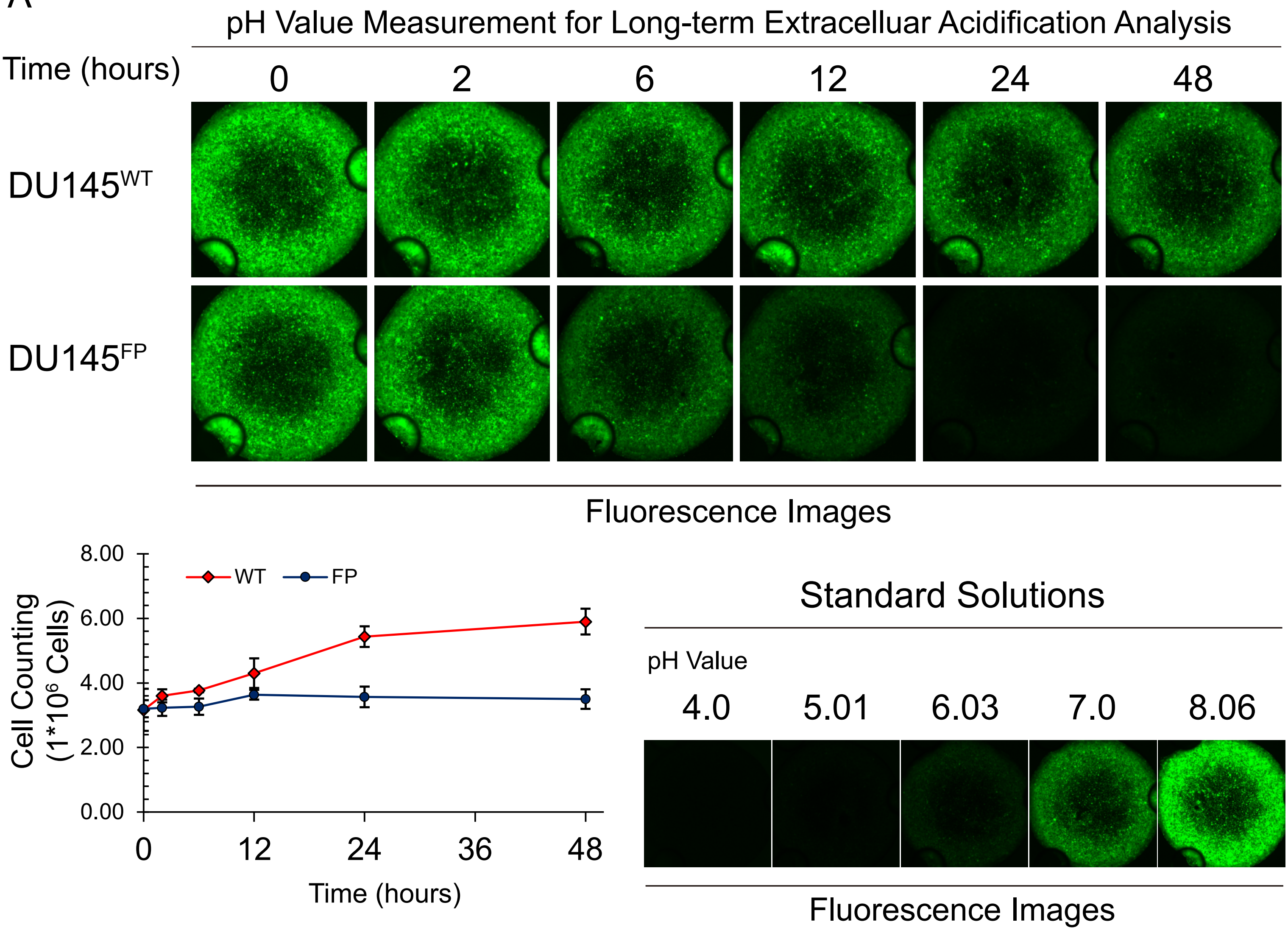

B

Color Index Log_2(Folds)				
-2	-1	0	1	2

CKIs

Gene Symbol	FP vs. WT	P	FDR
CDKN2B($P15^{INK4B}$)		***	0.00
CDKN1A($P21^{CIP1}$)		***	0.00
CDKN3(Cip2)		***	0.00
CDKN2D($P19^{INK4D}$)		NS	0.64
CDKN2C($P18^{INK4C}$)		NS	0.82
CDKN2A($P16^{INK4A}$)		NS	0.12
CDKN1B($P27^{KIP1}$)		***	0.00
CDKN1C($p57^{Kip2}$)		NS	0.35

CDKs

Gene Symbol	FP vs. WT	P	FDR
CDK6		***	0.00
CDK19		***	0.00
CDK5		***	0.00
CDK7		***	0.00
CDK11A		***	0.00
CDK2		***	0.00
CDK16		***	0.00
CDK11B		*	0.05
CDK13		*	0.06
CDK1		***	0.00
CDK17		***	0.01
CDK9		***	0.00
CDK18		***	0.00

Cyclin

Gene Symbol	FP vs. WT	P	FDR
CCNA1		***	0.00
CCNE2		***	0.00
CCNA2		***	0.00
CCNO		***	0.00
CCND1		***	0.00
CCND3		***	0.00
CCNY		***	0.00
CCNE1		**	0.01
CCNG1		***	0.00
CCNI		***	0.00
CCNL2		**	0.02
CCNB1		*	0.06
CCNL1		**	0.01
CCNK		***	0.00
CCNB3		*	0.09
CCNF		***	0.00

Figure S8

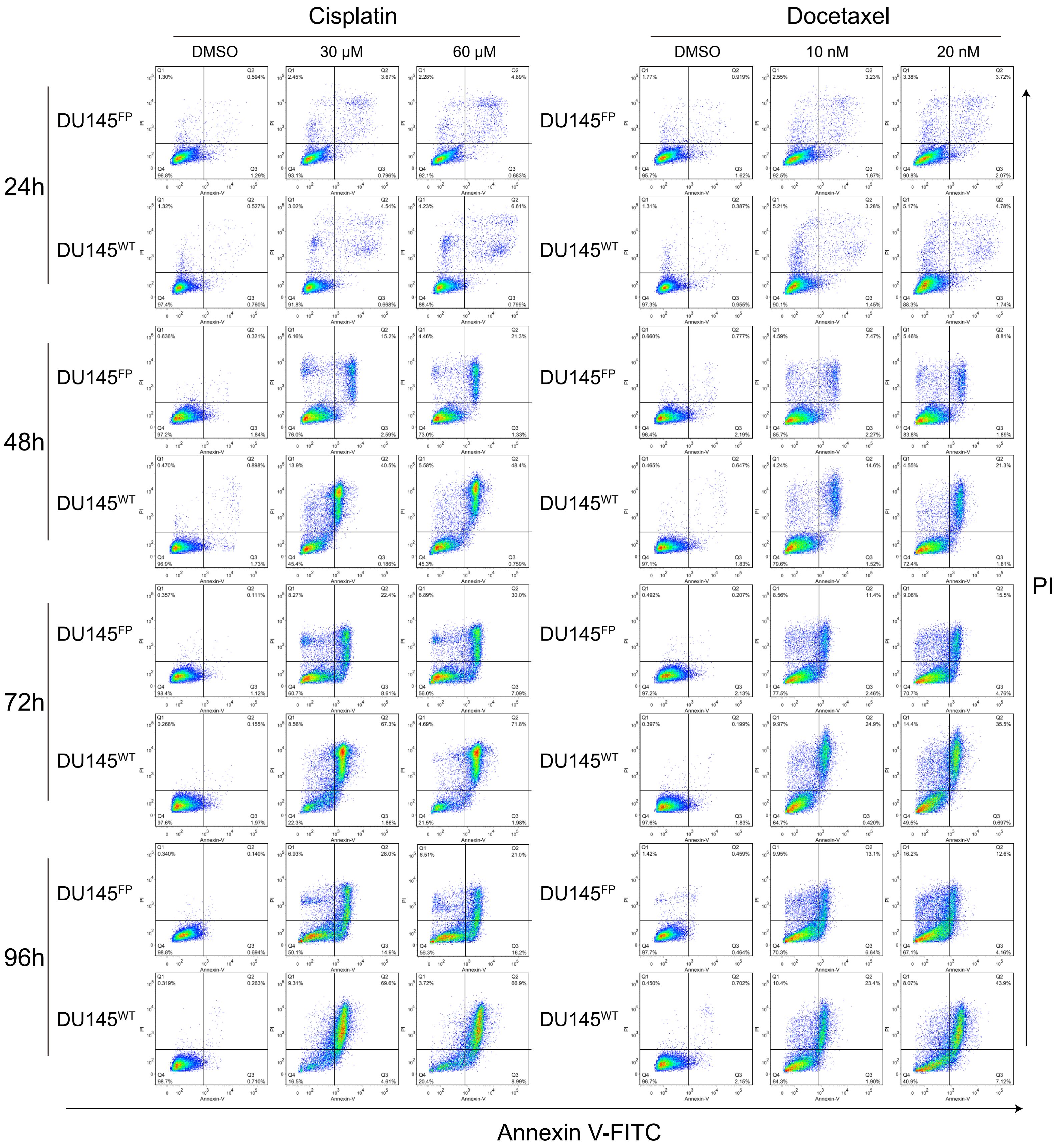

Cisplatin
Docetaxel
DMSO
30 μM
60 μM
DMSO
10 nM
20 nM
24h
48h
72h
96h
DU145FP
DU145WT
PI
Annexin V-FITC

Figure S9

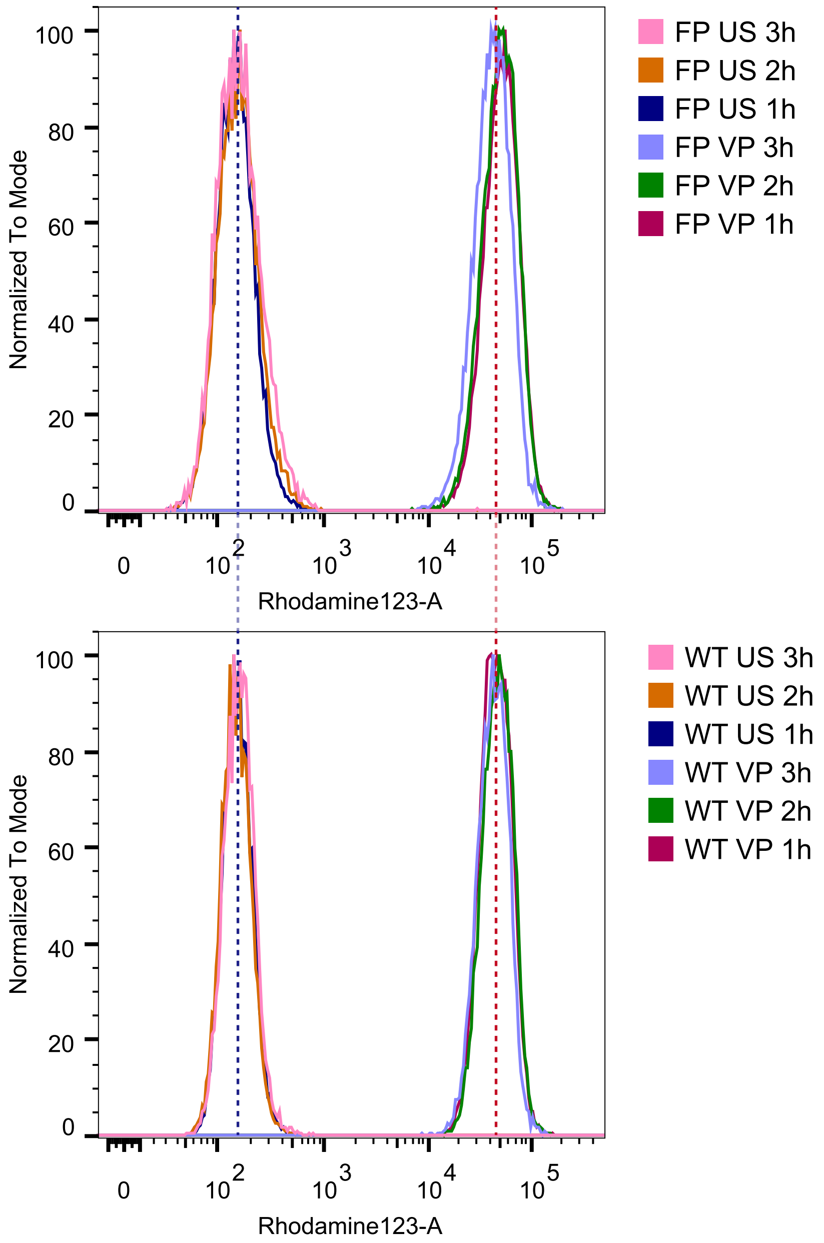
Normalized To Mode
100
80
60
40
20
0
0
10^2
10^3
10^4
10^5
Rhodamine123-A
FP US 3h
FP US 2h
FP US 1h
FP VP 3h
FP VP 2h
FP VP 1h
Normalized To Mode
100
80
60
40
20
0
0
10^2
10^3
10^4
10^5
Rhodamine123-A
WT US 3h
WT US 2h
WT US 1h
WT VP 3h
WT VP 2h
WT VP 1h

Figure S10

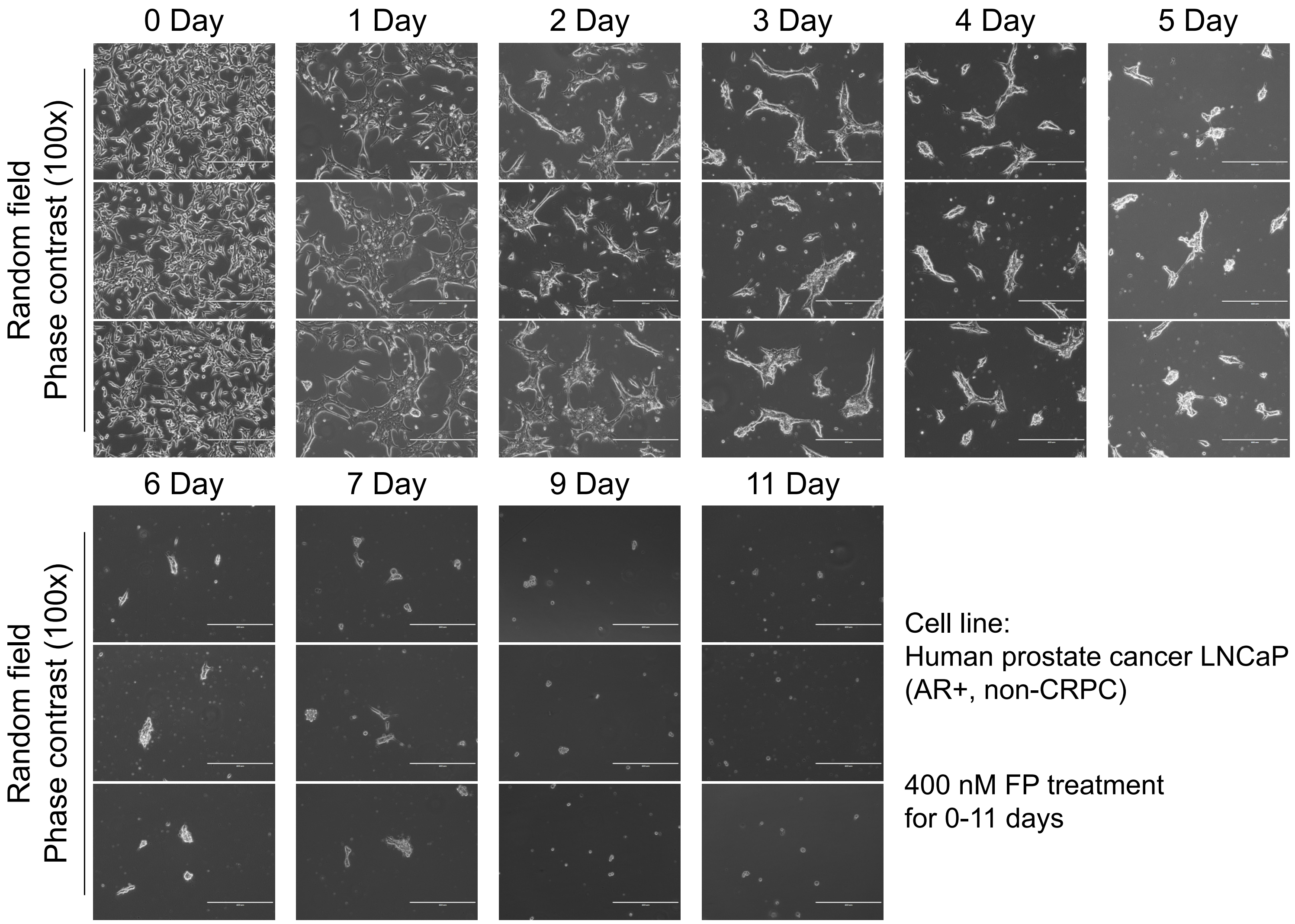
0 Day
1 Day
2 Day
3 Day
4 Day
5 Day
6 Day
7 Day
9 Day
11 Day
Random field
Phase contrast (100x)
Random field
Phase contrast (100x)
Cell line:
Human prostate cancer LNCaP
(AR+, non-CRPC)
400 nM FP treatment
for 0-11 days

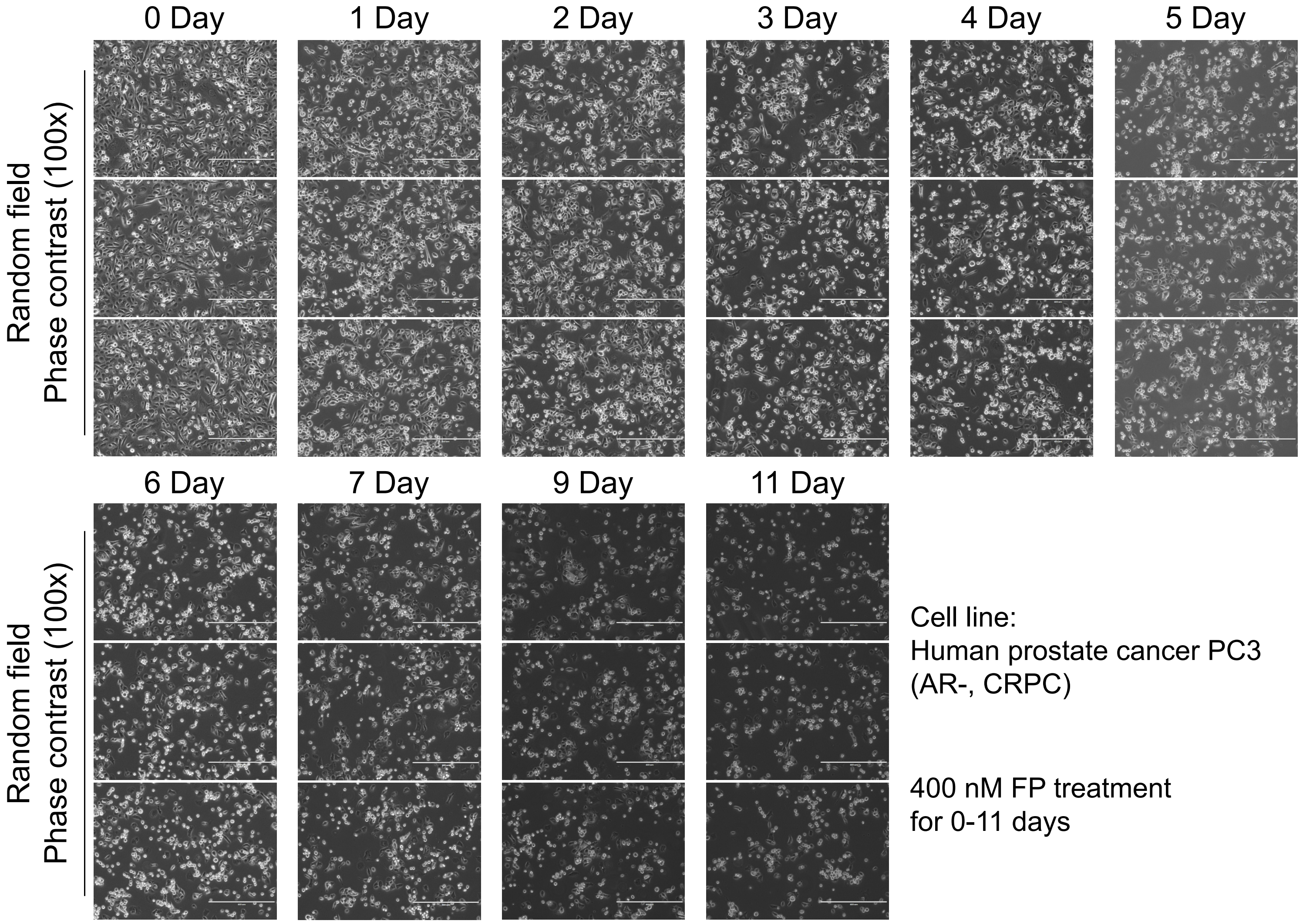
0 Day
1 Day
2 Day
3 Day
4 Day
5 Day
6 Day
7 Day
9 Day
11 Day
Random field
Phase contrast (100x)
Random field
Phase contrast (100x)
Cell line:
Human prostate cancer PC3
(AR-, CRPC)
400 nM FP treatment
for 0-11 days

Figure legends for supplemental figures and videos.

Figure S1. DU145^{FP} cells tolerate the treatment even with 10-folds higher FP concentration.

(A) The cells were treated with 400-4000 nM FP for durations of 24-96 hours and the dead cells were detected via flow cytometry by staining the cells with sufficient PI reagent. (B) The cell viabilities were summarized and shown in the line charts.

Figure S2. FP induced fast mitochondrial depolarization effect in the DU145 cells. The DU145 cells were treated with 400 nM FP for 2-48 hours, and mitochondrial membrane potential of the cells was evaluated by using flow cytometry. The x-axis represent fluorescence intensity of JC-1 monomers, and the y-axis represent fluorescence intensity of JC-1 aggregates.

Figure S3. The altered expression of ETC related genes and the increased ROS production in DU145^{FP} cells. (A) Transcriptome analysis of ETC related genes and mtDNA encoded genes The Log_2 transformed ratio of FPKM values (e.g., FP/WT) are indicated by color-coded index bars. (B) The cells' intracellular ROS level was analyzed via flow cytometry by staining the cells with sufficient mitochondrial ROS probe MitoSOX™ Red reagent. The left diagrams show typical flow cytometry analyses, and the right histograms show the relative MitoSOX™ Red fluorescence intensities, which represent the geometric mean values of the full fluorescence signal area of MitoSOX red probe. The data is presented as means from three independent experiments (mean ± S.D.).

Figure S4. FP sensitivity of serum starvation-induced slow cycling DU145 cells.

(A) The DU145^{WT} cells show slow cycling when cultured in serum free medium without FP for three days (WT S-F-), but the total cell number of the DU145 cells cultured in serum free medium with FP (WT S-F+) shows almost no change. Comparatively, the DU145^{WT} cells cultured in the medium with serum (WT S+F+) were less sensitive to FP (red line). But the most FP resistant cells were the DU145^{FP} cells, which revealed no big difference when cultured in the medium with or without serum (light blue and yellow lines). The line charts show the cell proliferation kinetics based on the cell counting results at day 0, day 1 and day 3. (B) The cell viabilities on day 1 and day 3 were assessed by using flow cytometry and APC conjugated Annexin-V/7-AAD double staining assay. The double negative cells were considered as viable, and the histograms represent the percentages of viable cells. The data is presented as means from three independent experiments (mean ± S.D.). (C) A group of representative results of the analyses were shown.

Figure S5. Transcriptome analysis of anti-apoptosis and pro-apoptosis genes. The Log_2 transformed ratio of FPKM values (e.g., FP/WT) are indicated by color-coded index bars. The aliases of genes are summarized and shown on the left.

Figure S6. An overview of FP influenced mitochondrial controlled apoptosis pathways. The annotations can be found in the lower right corner. For convenient, the transcriptome data are shown aside of each regulator as paired circles with color filled, and the last number/character of each gene isotype is shown on the left close to the circle pairs correspondingly.

Figure S7. Verification of extracellular acidification, slow cycling and cell cycle-related gene expressions in FP treated DU145 cells. (A) The extracellular acidification ratios of the cells were evaluated by monitoring the pH value fluctuation of non-buffered Seahorse DMEM medium for 0-48 hours cell culturing. The images display fluorescence intensities of the pH sensors under excitation state. The upper row displays the sensor images for cell culture medium collected at 0-48 hours. The lower right row shows the sensor images for pH standard solutions. The related cell counting results are shown in the line charts lower left. (B) Transcriptome analysis of CDKs, CKIs and cyclin genes. The Log_2 transformed ratio of FPKM values (e.g., FP/WT) are indicated by color-coded index bars.

Figure S8. Drug sensitivity evaluation by flow cytometry detection of apoptotic cells. The wild type and FP resistant DU145 cells were treated with cisplatin (30 µM and 60 µM) and docetaxel (10 nM and 20 nM) for 24-96 hours. For each 24 hours, the apoptotic cells were detected by using flow cytometry and FITC conjugated Annexin V/PI double staining assay. Representative diagrams of the experiment show reduced sensitivity of $DU145^{FP}$ cells to cisplatin and docetaxel treatments.

Figure S9. The unstained cells and verapamil (VP) negative control cells for Rhd-123 efflux assays. The Rhd-123 fluorescence intensities of the cells stained with Rhd-123 with additional verapamil (VP) and unstained cells (US) are shown in diagrams as reference. The red dashed lines were normalized to the center of the fluorescence signals of wild type cells.

Figure S10. Both PC3 and LNCaP prostate cancer cells are FP sensitive. LNCaP and PC3 cells were treated with 400 nM FP for 11 days, and the cell density and morphological changes of the cells were monitored by daily captured images (phase contrast). The scale bar equals to 400 µm.

Video SV1. Enhanced cell motility during the 400 nM treatment of FP. This video shows the status of DU145 cells during the FP treatment starting from day 8 to day 10. The DU145 cells around this period are frequently found in syncytia and with high motility. One second in the video equals to 25 minutes of real time.

Video SV2. The proliferation manner of the wild type DU145 cells. This video shows the status of wild type DU145 cells in the medium without FP after being passaged. Wild type DU145 cells have a relatively uniformed morphology, and are fast dividing. One second in the video equals to 25 minutes of real time.

Video SV3. Wound healing assay. This video shows a comparison of representative healing assays. The $DU145^{FP}$ cells are morphologically larger, having lower density, but faster migration during wound healing process than the wild type DU145 cells. One second in the video equals to 25 minutes of real time.
